# The plant hormone, 6-benzylaminopurine, ameliorates obesity in male and female mice while on a high-fat diet

**DOI:** 10.1016/j.molmet.2026.102366

**Published:** 2026-04-11

**Authors:** Calvin V. Lieu, Cindy X. Zhang, Neruja Loganathan, Leon French, Andre Krunic, Sarah B. Cash, Jin Shi, Juliette Lee, Kacey J. Prentice, Carolyn L. Cummins, Jesse Gillis, Denise D. Belsham

**Affiliations:** 1 Department of Physiology, Temerty Faculty of Medicine, University of Toronto, Toronto, Canada; 2 Department of Pharmacology, Physiology, and Biophysics, Boston University Chobanian and Avedisian School of Medicine, Boston, USA; 3 Department of Pharmaceutical Sciences, Leslie Dan Faculty of Pharmacy, University of Toronto, Toronto, Canada; 4 Banting and Best Diabetes Centre, University of Toronto, Toronto, Canada; 5 Department of Medicine, Temerty Faculty of Medicine, University of Toronto, Toronto, Canada

**Keywords:** Obesity, Weight loss, Appetite suppression, Hypothalamus, Neuropeptide Y, MEK/ERK, EGR1, Phytohormone

## Abstract

Obesity is a global health crisis. Currently available treatments, while effective, show several undesirable side effects that hinder their long-term use. Herein, we investigated the anti-obesity potential of 6-benzylaminopurine (BAP), a plant hormone commonly used in agricultural settings to enhance plant development, in obese mice and mammalian cell models. Orally administered BAP induced significant weight loss in diet induced obese male and female CD-1 mice through sex-specific mechanisms involving appetite suppression, adipose tissue remodeling, and enhanced lipid utilization. Concurrently, BAP improves several metabolic parameters associated with obesity, including glucose tolerance, fasting blood glucose, hyperleptinemia, hyperinsulinemia, white adipose tissue browning, and liver health. In murine- and human-hypothalamic neuronal models, BAP suppresses the expression of feeding stimulating neuropeptide Y (*Npy*) and increases the anorexigenic pro-opiomelanecortin (*Pomc*). Using RNA-sequencing, we identified that BAP inhibits EGFR/ErbB2 and MEK/ERK/EGR1 signaling, whereas MEK/ERK inhibition is partially responsible for the *in vitro* effects of BAP, including *Npy* downregulation. Moreover, similar MEK/ERK inhibition was also shown to be involved in the induction of thermogenic markers, including uncoupling protein 1 (*Ucp1*), in 3T3-L1 derived adipocyte, indicating a consistent molecular mechanism of BAP across different cell types. Overall, our data showed that BAP could serve as an efficacious and alternative treatment avenue for obesity with a unique mechanism of action compared to currently available options.

## Highlights

• The phytohormone BAP caused weight loss in obese mice via sex-specific mechanisms.
• BAP suppressed food intake in male mice but not in females.
• BAP enhanced fat utilization and white adipocytes browning markers in female mice.
• BAP reduced the orexigenic neuropeptide Y in murine- and human-hypothalamic models.
• BAP reduced neuropeptide Y by inhibiting MEK/ERK/EGR1 in the mHypoE-46 cells.

## Graphical abstract

**Figure:**
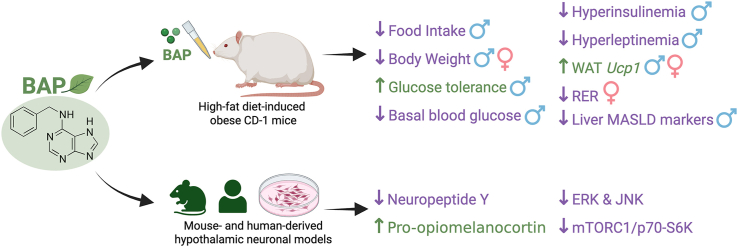

## 1 Introduction

Obesity is a global health crisis that has been estimated to affect 1 in 8 people worldwide [1]. With an updated definition, including body mass index (BMI), anthropometric measurements, and excess adiposity, an additional 26% of adults are estimated to live with obesity and are at risk of comorbidities, including type 2 diabetes mellitus (T2DM), cardiovascular disease, and certain cancers [[2], [3], [4], [5], [6], [7]]. The advent of glucagon-like peptide-1 receptor (GLP-1R) agonist-based therapies, such as semaglutide and tirzepatide, have provided effective pharmaceutical options for many obese patients due to their ability to induce significant weight loss and improve obesity associated comorbidities, such as insulin resistance, hyperglycemia, and hyperlipidemia [8]. However, these drugs still have major side effects, such as gastrointestinal events, nausea, muscle atrophy, and decreased bone density, which hinder their long-term use [[9], [10], [11]]. As a result, only 31% of patients remain on these drugs after a year, and those who discontinue the drug regain two-thirds of the weight they lost within a year [12,13]. Additionally, a 2021 phase 3 randomized clinical trial assessing the weight loss efficacy of semaglutide showed that ∼14% of treatment recipients achieved less than 5% weight loss after 68 weeks, which is below the clinical threshold for non-response [14]. Thus, there remains a need for alternative and innovative obesity therapeutics, as well as maintenance options for weight loss.

**Figure 1:**
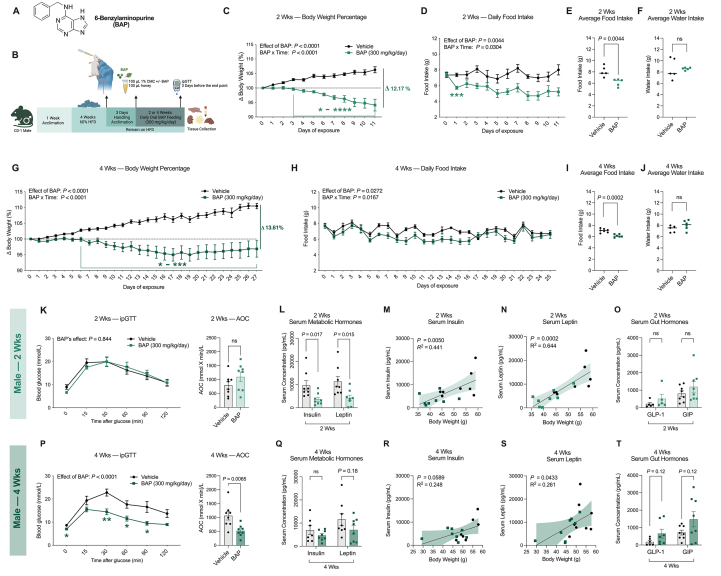
**BAP decreases food intake and body weight and improves glucose homeostasis in HFD-fed male CD-1 mice**. **(A)** The chemical structure of BAP. **(B)** Experimental outline for the male mice cohorts. 7-week-old male CD-1 mice were acclimated with a standard chow diet in the animal facility for 1 week and were fed a HFD for 4 weeks to induce an obese phenotype (DIO). All mice received 3 days of emulsion feeding acclimation, followed by 2 or 4 weeks feeding of BAP (300 mg/kg/day) or vehicle emulsion. ipGTT was conducted in a subset of mice 3 days before the experimental endpoint. **C–F,** Changes in **(C)** body weight as a percentage of starting weight, **(D)** daily food intake, **(E)** average daily food intake per cage of 2 mice, and **(F)** average daily water intake per cage of 2 mice fed with BAP or vehicle emulsion for 2 weeks (*n* = 10 for body weight measurements, *n* = 5 for food and water intake measurements as per cage of 2 mice). **G-J,** Changes in **(G)** body weight as a percentage of starting weight, **(H)** daily food intake, **(I)** average daily food intake per cage of 2 mice, and **(J)** average daily water intake per cage of 2 mice fed with BAP or vehicle emulsion for 4 weeks (*n* = 16 for body weight measurements, *n* = 8 for food and water intake measurements per cage of 2 mice). **K-T,** Before the experimental endpoint, a subset of mice from each cohort were randomly selected for ipGTT (*n* = 8). Glucose tolerance and area of curve (AOC = area of curve) measurements of CD-1 mice after **(K)** 11 days or **(P)** 25 days of BAP or vehicle emulsion feeding (*n* = 8). Serum concentration of insulin and leptin of CD-1 mice fed with BAP or vehicle emulsion for **(L)** 2 weeks or **(Q)** 4 weeks (*n* = 8). Serum levels of **(M, R)** insulin and **(N, S)** leptin were correlated with the final body weight of each mouse using simple linear regression with 95% confidence bands around the best-fit line (*n* = 8). Serum concentration of GLP-1 and GIP of CD-1 mice fed with BAP or vehicle emulsion for **(O)** 2 weeks or **(T)** 4 weeks (*n* = 8). Data were analyzed using two-way ANOVA with Bonferroni post-hoc test for all daily measurements of body weight percentage and food intake **(C, D, G, H)** and glucose tolerance **(K, P).** Unpaired two-tailed *t*-test was used for 2 group comparisons **(E, F, I, J, K, L, O, P, Q, T)**. Values are presented as mean ± SEM with *P* values. *P*-values greater than 0.2 are presented as ns. Δ represent the net differences in body weight percentage between BAP and vehicle groups. Days or time points that show significant differences between BAP and vehicle groups for body weight **(C, G)**, food intake **(D)** and blood glucose **(P)** are presented with ∗ *P* < 0.05, ∗∗*P* < 0.01, ∗∗∗*P* < 0.005, ∗∗∗∗*P* < 0.0001.

Phytohormones, also referred to as plant hormones, are specialized signaling molecules that are endogenously produced by plants at low concentrations (nM) to regulate plant physiology [[15], [16], [17]]. These characteristics make phytohormones different from plant produced compounds, like resveratrol and caffeine, which are produced in greater quantities and serve structural, defensive, or ecological roles without inherent hormonal signaling functions [18]. The ubiquitous phytohormone, 6-benzylaminopurine (N^6^-benzyladenine/BAP), is an adenine-derived cytokinin that regulates plant growth and development (Figure 1A) [19,20]. Currently, BAP is commonly used in plant cell culture and in commercial settings to extend the lives of produce and flowers, and it has been approved by the US Environmental Protection Agency (EPA) for agricultural use as a plant growth regulator in 1997 [21]. Emerging studies indicate that related phytohormones can also work in mammalian systems and hold therapeutic potential to treat human diseases [22], but BAP has not yet been studied in humans. The best-known example of related phytohormones are phytoestrogens (e.g., soy), which are antimicrobials in plants but can interact with mammalian estrogen receptors and have anti-inflammatory and cholesterol-lowering effects in humans [[23], [24], [25], [26]]. In plants, cytokinins like BAP are postulated to signal through histidine kinases, but no homologous receptor system has been described in mammals; thus, their effects and mechanisms of action in animals remain unknown [27]. Yet, BAP and kinetin, another member of the cytokinin subfamily, have been previously shown to act as anti-proliferative agents in tumour and fibroblast cells and antioxidants in skin fibroblasts [28,29]. Moreover, kinetin has undergone clinical trials for the treatment of familial dysautonomia and is being investigated for treating Huntington’s disease, suggesting the potential of cytokinins to exert biological benefits in mammalian models [30,31]. When evaluating the safety of BAP for agricultural use, in 1994, the EPA conducted a range of toxicity studies in rats, rabbits, and beagle dogs, assessing its safety for long-term oral ingestion (up to 90 days), reproduction, chromosomal effects, and gene mutation [32]. While no significant adverse effects were documented in these categories, the EPA report briefly mentioned a weight loss effect of BAP in rats with no supporting data provided [32]. Since then, studies testing the effects of BAP in animals or mammalian cells are limited, with most focus on zebrafish [[33], [34], [35], [36]]. The weight-loss potential of BAP, as well as its effect on metabolic health in mammalian models, has not been explored. Thus, after promising pilot experiments in neuronal cell models, we decided to investigate the potential use of BAP for treating obesity and associated comorbidities in obese mouse models.

Obesity is primarily caused by a positive energy balance, wherein energy intake consistently exceeds energy expenditure. This balance, and ultimately body weight, is meticulously coordinated by the arcuate nucleus (Arc) of the hypothalamus, where the blood brain barrier is permeable, allowing the integration of circulating nutrient and hormonal signals to regulate feeding and energy expenditure accordingly [37]. The Arc controls energy homeostasis via two major neuronal subpopulations, the orexigenic *neuropeptide Y (Npy)* and *agouti-related peptide* (*Agrp*)-expressing neurons and the anorexigenic *pro-opiomelanecortin* (*Pomc*)-expressing neurons [37]. These first-order *Npy/Agrp*- and *Pomc*-expressing neurons directly sense circulating signals through a semi-permeable median eminence and secrete respective neuropeptides that act on second-order neurons in other hypothalamic nuclei, including the paraventricular hypothalamic nucleus (PVN), dorsomedial hypothalamus (DMH), lateral hypothalamus (LH), and the ventromedial hypothalamus (VMH) [38,39]. The second-order neurons further convey the received information and project to regions outside the hypothalamus, such as the nucleus tractus solitarius (NTS) and the parabrachial nucleus (PBN) in the brainstem, to adjust feeding, energy expenditure, and thermogenesis, accordingly [[40], [41], [42], [43]]. Meanwhile, peripheral appetite regulators, such as insulin and amylin from the pancreas, GLP-1 and glucose-dependent insulinotropic polypeptide (GIP) from the gut, fibroblast growth factor 21 (FGF21) from the liver, and leptin from the adipose tissue, fluctuate based on energy status, and signal to the brain via hormonal and neuronal (via vagal afferents to brainstem) routes, often converging on the hindbrain and hypothalamic circuit [8,[44], [45], [46], [47], [48], [49], [50], [51], [52], [53]]. Together, this bidirectional brain-peripheral axis regulates feeding and energy homeostasis collaboratively, whereas dysfunction in any component of the neuroendocrine apparatus can lead to energy imbalance. In the obese state and upon HFD consumption, the hypothalamic neurons become resistant to peripheral satiety signals, ultimately altering the expression of *Npy, Agrp*, and *Pomc,* further disrupting the neuroendocrine communication and exacerbating obesity [54,55].

In this study, we report the efficacy of BAP in causing significant weight loss and ameliorating obesity-associated metabolic dysfunction in high fat diet (HFD)-fed male and female CD-1 mice, via mechanisms involving appetite suppression and enhanced lipid utilization as energy. In hypothalamic neuronal models, BAP induces an anorexigenic response by decreasing the potent orexigenic neuropeptide *Npy*, partially through inhibiting the mitogen-activated protein kinase (MAPK)/extracellular signal-regulated kinase (ERK1/2)/early growth response protein 1 (EGR1) signaling cascade. Moreover, MEK/ERK inhibition blocked the effect of BAP on the induction of uncoupling protein 1 (*Ucp1*), a key thermogenesis and browning marker, in 3T3-L1 derived adipocyte cells. Overall, we have identified a plant hormone that holds promise as an alternative, efficacious treatment for obesity with unique mechanisms in mammalian models.

## 2 Results

### 2.1 Orally administered BAP decreases body weight and improves glucose tolerance in male HFD-fed mice

To investigate the efficacy of BAP *in vivo*, male CD-1 mice were maintained on a 60% kilocalories from fat, high fat diet (HFD), for 4 weeks, resulting in an obese phenotype. The mice were then orally administered BAP daily at a dose of 300 mg/kg (chosen after a dose curve (Fig. S1A–S1C)) using a voluntary honey emulsion feeding protocol for 2 or 4 weeks while remaining on *ad-libitum* HFD feeding (Figure 1A, B) [32,56]. Male mice fed BAP for 2 weeks showed a 5% reduction in body weight, while the vehicle group continued to gain weight, resulting in a final body weight difference of 12.17% (Figure 1C, Fig. S1D). Notably, the decreased body weight in BAP-fed mice was accompanied by an immediate reduction in daily food intake, as early as one day of BAP exposure, resulting in a 20% decrease in daily average food intake compared to the vehicle group after 2 weeks (Figure 1D, E, Fig. S1B). No changes in water intake were observed (Figure 1F). In the 4-week cohort, BAP-fed mice achieved a maximum weight loss of 5% after 19 days and maintained this state for the remainder of the exposure period, whereas vehicle-fed mice continued to gain weight (Figure 1G, Fig. S1E). Like the 2-week cohort, a 13% reduction in average daily food intake was observed in BAP-fed mice with no change in water intake, suggesting that reduced body weight is likely caused by BAP mediated appetite suppression (Figure 1H–J).

In addition, we assessed whether BAP improves glucose homeostasis in these mice using an intraperitoneal glucose tolerance test (ipGTT). Mice fed BAP for 4 weeks displayed significantly decreased fasting blood glucose levels, along with improved glucose tolerance (Figure 1P). These improvements were not observed in the 2-week cohort, suggesting that the beneficial effects on glucose homeostasis are likely caused by sustained weight loss by BAP instead of its direct actions (Figure 1K). In contrast, serum insulin and leptin levels were significantly lower in mice fed BAP for 2 weeks, but not 4 weeks (Figure 1L, Q). In both cohorts, serum insulin and leptin levels were positively correlated with body weight, implying these changes in serum hormones are likely a direct consequence of BAP-mediated weight loss (Figure 1M–S). In addition, we measured the circulating levels of gut hormones due to their role in appetite regulation. Under non-fasting conditions, we observed a trend towards increased levels of endogenous serum GIP and GLP-1 by 177% and 391%, respectively, at 4 weeks but not 2 weeks (Figure 1O, T). Overall, we found that oral BAP feeding decreased body weight, suppressed food intake, and reduced hyperinsulinemia and hyperleptinemia in obese male CD-1 mice.

### 2.2 BAP increases WAT browning markers and decreases hepatic MASLD markers in male HFD-fed mice

**Figure 2:**
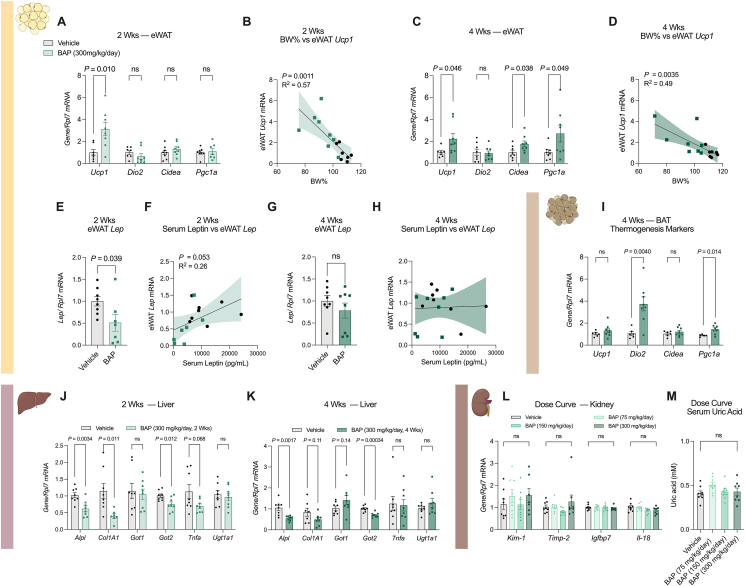
**BAP increases WAT thermogenic markers and decreases hepatic MASLD markers in male CD-1 mice**. **A-D,** The effect of daily BAP feeding on the mRNA expression of thermogenic markers, *Ucp1, Dio2, Cidea, Pgc1a*, in eWAT of male CD-1 mice after **(A)** 2 weeks or **(C)** 4 weeks of 300 mg/kg/day BAP (*n* = 8). Relative mRNA levels of *Ucp1* were correlated with the body weight of each mouse from the **(B)** 2 and **(D)** 4-week male cohorts using simple linear regression with 95% confidence bands around the best-fit line (*n* = 8). **E-H,** Changes in eWAT *Lep* mRNA expression in male CD-1 mice following **(E)** 2 or **(G)** 4 weeks of 300 mg/kg/day BAP feeding (*n* = 8). Relative *Lep* mRNA expression in eWAT was correlated with serum leptin levels of male **(F)** 2 week and **(H)** 4-week 300 mg/kg/day BAP cohorts (*n* = 8). **I,** The effects of 4-week BAP on the mRNA expression of thermogenic markers in BAT of male CD-1 mice. **J,K,** The effect of daily BAP feeding on mRNA expression of liver function markers, *Alpl, ColA1, Got1, Got2, Tnfa, Ugt1a1*, in the liver of male CD-1 mice after **(J)** 2 weeks or **(K)** 4 weeks of 300 mg/kg/day BAP (*n* = 8). **L,M, (L)** The effect of daily BAP feeding on the mRNA expression of kidney function markers, *Kim-1, Timp2, Igfbp7,Il-18*, in the kidney, and **(M)** serum uric acid levels, in CD-1 mice after 2 weeks of 75, 150, 300 mg/kg/day BAP (*n* = 8). Data was analyzed using unpaired two-tailed *t*-test. Values are presented as mean ± SEM with *P* values. *P*-values greater than 0.2 are presented as ns.

Next, we wanted to explore the impact of BAP on peripheral organs that are often influenced by obesity, including white adipose tissue (WAT), brown adipose tissue (BAT), and the liver. WAT browning has emerged as a promising target for weight loss by shifting white adipocyte from an energy storage phenotype into a beige/brown adipocyte-like state that dissipates energy as heat. However, chronic systemic inflammation caused by obesity impairs WAT browning and beige adipocyte development, hindering their thermogenic capacity and exacerbating adipose tissue dysfunction [57,58]. To assess whether BAP could alter WAT browning, we measured several markers of browning and thermogenesis in the epididymal WAT (eWAT). BAP significantly upregulated the mRNA expression of *Ucp1*, the primary marker of thermogenesis, by 213% and 123% in the eWAT of male mice subjected to 2- and 4-weeks of BAP, respectively (Figure 2A, C). Moreover, other browning markers such as cell death inducing DFFA-like effector a (*Cidea*) and PPARG coactivator 1 alpha (*Pgc1a*) were also increased in male mice after 4 weeks of BAP feeding (Figure 2C). Interestingly, eWAT *Ucp1* expression was strongly and negatively correlated with body weight change upon BAP exposure, as mice with higher eWAT *Ucp1* levels displayed stronger BAP-mediated weight loss, suggesting the potential involvement of WAT browning in the weight loss effect of BAP (Figure 2B, D). Concurrently, we assessed leptin (*Lep*) expression in eWAT, as WAT is the main leptin-producing tissue, and BAP decreased HFD-induced hyperleptinemia in these mice. Male mice fed BAP for 2 weeks displayed significantly lower eWAT *Lep* mRNA expression that positively correlated with serum leptin levels (Figure 2E, F). Consistent with what we observed in the serum, eWAT *Lep* remained unchanged by BAP at 4 weeks, suggesting that the improved hyperleptinemia may be due to BAP influencing leptin production capacity in WAT (Figure 2G, H). In parallel, BAP significantly increased the expression of type 2 iodothyronine deiodinase (*Dio2*) and *Pgc1a* in the interscapular BAT in the 4-week cohort, implying potential enhancement in thermogenic capacity (Figure 2I).

The liver is the primary tissue for fatty acid metabolism and plays a key role in glucose homeostasis. Excessive dietary fat consumption promotes the development of metabolic dysfunction-associated steatosis liver disease (MASLD), wherein ectopic hepatic lipid accumulation can result in liver fibrosis and failure [59]. We measured a selection of common markers associated with liver function and MASLD, whereby BAP significantly decreased metabolism enzymes *alkaline phosphatase (Alpl)* and *glutamic-oxaloacetic transaminase 2* (*Got2*), which are typically elevated in MASLD, after 2 and 4 weeks of BAP feeding in the male mice (Figure 2J, K). Furthermore, a significant decrease in tissue fibrosis marker *collagen alpha 1 (Col1a1)* was detected after 2 weeks BAP feeding, suggesting improved liver health (Figure 2J). Overall, BAP-fed mice show lowered hepatic MASLD markers, which can be attributed to a reduction in HFD feeding diminishing ectopic fat accumulation in the liver.

Lastly, due to structural similarities between BAP and adenine, we considered the possibility that BAP may be metabolized to adenine and subsequently converted to uric acid, which could result in kidney stones. However, BAP did not increase serum uric acid levels, nor the expression of multiple kidney function markers measured (Figure 2L, M).

### 2.3 BAP induces weight loss in female HFD-fed mice independent of appetite regulation

**Figure 3:**
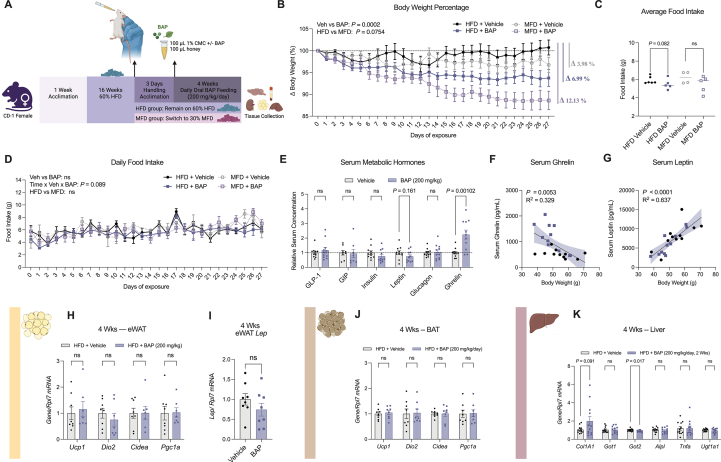
**BAP decreases body weight without changing food intake in HFD-fed female CD-1 mice**. **A,** Experimental outline for the female mice cohorts. 7-week-old female CD-1 mice were acclimated with a standard chow diet in the animal facility for 1 week and were then fed a HFD for 16 weeks to induce an obese phenotype. All mice received 3 days of emulsion feeding acclimation, and a subset of mice were switched to MFD during this time while the rest remained on HFD. All mice were then randomly assigned to be fed BAP (200 mg/kg/day) or vehicle emulsion for 4 weeks. **B-D,** Changes in **(B)** body weight as a percentage of starting weight, **(C)** average food intake, and **(D)** daily food intake per cage of 2 mice of female CD-1 mice fed 200 mg/kg/day BAP or vehicle emulsion for 4 weeks, with or without MFD intervention (*n* = 12 for HFD groups, *n* = 8–9 for MFD groups). **E-J**, Relative serum concentration of metabolic hormones of **(E)** female CD-1 mice fed with BAP (200 mg/kg/day, 4 weeks) or vehicle emulsion without MFD intervention (*n* = 11). Values were normalized to vehicle. Body weight was correlated to serum concentration of **(F)** ghrelin and **(G)** leptin in female mice using simple linear regression with 95% confidence bands around the best-fit line (*n* = 11). **H, I,** Changes in the mRNA expression of **(H)** thermogenic markers and **(I)***Lep* in WAT of female mice after 4 weeks of 200 mg/kg/day BAP (*n* = 11). **J, K,** Changes in the mRNA expression of **(J)** thermogenic markers in BAT and **(K)** liver health markers in the liver of female mice after 4 weeks of 200 mg/kg/day BAP (*n* = 11). Data were analyzed using three-way ANOVA with Bonferroni post-hoc test for daily measurements of body weight percentage and food intake in the HFD vs MFD experiments **(B, D)**. Two-way ANOVA with Bonferroni post-hoc test was used for average body percentage weight comparison **(B).** Unpaired *t-tests* were used to compare serum hormones and tissue markers between BAP and vehicle-fed groups **(E, H–K).** Values are presented as mean ± SEM with *P* values. *P*-values greater than 0.2 are presented as ns. Δ represent the net differences in body weight percentage between the BAP group and the vehicle control group.

We then questioned whether BAP could induce comparable metabolic benefits in females. Female CD-1 mice were provided *ad*-*libitum* HFD feeding for 16 weeks, as female mice take longer to develop an obese phenotype [[60], [61], [62]]. The mice were then fed an emulsion of BAP at a dose of 200 mg/kg or vehicle daily for 4 weeks (Figure 3A). Sex differences in drug response and sensitivity have been robustly reported across a variety of medications and thus accounting for these differences are essential for modeling clinically relevant scenarios [63]. Thus, we determined the dose for female mice based on the EPA report, which showed that female rats were more sensitive to the weight loss effects of BAP, starting at 121 mg/kg/day, whereas males required at least 295 mg/kg/day to observe weight loss [32]. BAP-fed female mice lost 6.3% of their initial body weight after 4 weeks of daily exposure while remaining on the HFD (Figure 3B, Fig. S1F). To mimic a clinically relevant weight management scenario, we also compared the effects of BAP with dietary intervention, whereas a subset of female mice was switched to a 30% kilocalorie from fat, medium fat diet (MFD), after 16 weeks of HFD feeding, combined with 200 mg/kg/day BAP or vehicle feeding for 4 weeks (Figure 3A). Interestingly, BAP produced a stronger decrease in body weight than diet intervention, which alone was ineffective to induce significant weight loss (Figure 3B). In the female mice that received BAP and MFD, an additive 12% reduction in body weight was observed after 4 weeks, showing that BAP could enhance weight loss efficacy alongside diet intervention in female mice. In contrast to the anorexigenic phenotype observed in male mice, no significant changes in food intake were detected in the females (Figure 3C, D). Therefore, the weight-loss effect of BAP in female mice is likely to be mediated by mechanisms independent of appetite.

Next, we assessed the effect of BAP on peripheral tissues and serum hormone levels. Female mice that were consistently maintained on the 60% HFD were used to eliminate potential confounding effects caused by diet intervention. When measuring the levels of circulating metabolic hormones, a significant increase in serum ghrelin was detected in female BAP-fed mice while other hormones remained unchanged (Figure 3E, Fig. S1G). Serum ghrelin levels were also negatively correlated with body weight change, suggesting it may be a compensatory mechanism in response to weight loss (Figure 3F). Although no significant change in serum leptin was detected, its levels were still positively correlated with body weight in the females, similar to the males (Figure 3G). No significant changes in circulating insulin, GLP-1, and GIP levels were observed with BAP feeding in female mice (Figure 3E, Fig. S1G). In the eWAT and BAT of female BAP-fed mice, no significant changes in browning markers or *Lep* were observed (Figure 3H–J). No changes in hepatic MASLD markers were found besides a significant but mild reduction in *Got2* mRNA expression (Figure 3K). However, since female mice are known to have delayed progression of diet induced MASLD, it is unclear whether the absence of improved liver health in the females was due to a sex-specific effect of BAP in the liver or these female mice have yet to develop MASLD [64].

### 2.4 BAP enhances lipid utilization and WAT function in a sex-specific manner in HFD-fed mice

**Figure 4:**
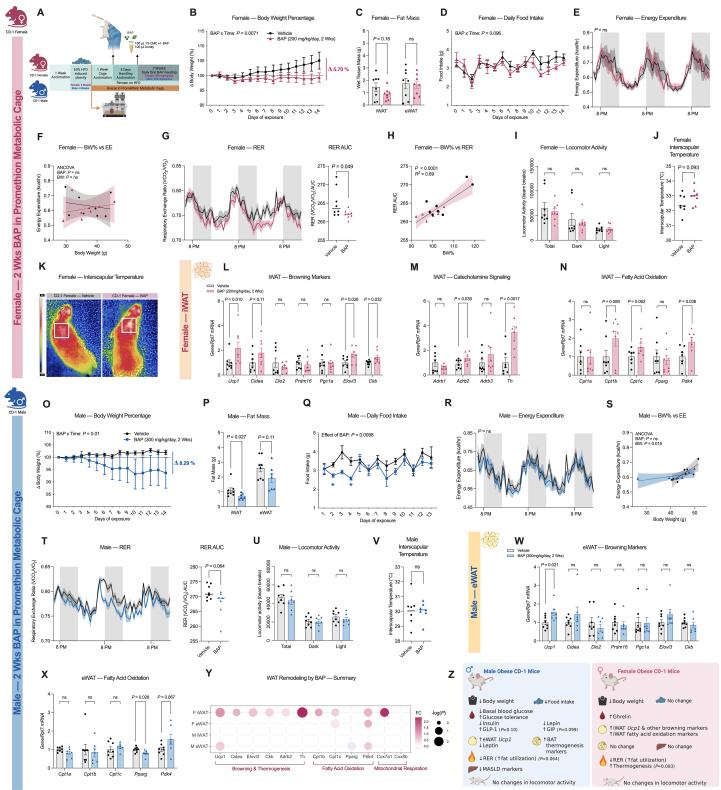
**BAP differentially alters RER and the mRNA expression of WAT function markers in female and male CD-1 mice**. **A,** Experimental outline for the metabolic cage experiment. 7-week-old female and male CD-1 mice were acclimated with a standard chow diet in the animal facility for 1 week and were then fed with HFD for 8 and 4 weeks, respectively, to induce an obese phenotype. All mice were moved into single-caged metabolic cage 1 weeks for cage acclimation and received 3 days of handling/emulsion feeding acclimation. All mice were then randomly assigned to be fed BAP (Female: 200 mg/kg/day, Male: 300 mg/kg/day) or vehicle emulsion for 2 weeks within the metabolic cage. **B-E,** Changes in **(B)** body weight as a percentage of starting weight, **(C)** fat tissue mass, **(D)** daily food intake, and **(E)** energy expenditure (EE) in female CD-1 mice fed 200 mg/kg/day BAP or vehicle emulsion for 2 weeks (*n* = 8). **F,** Regression plot of energy expenditure from female mice fed BAP or vehicle emulsion over the second week of the experiment (Day 8–14), *n* = 8. **G,** A representative fragment of the effects of BAP on respiratory exchange ratio (RER) in female CD-1 (left). The differences between total area under the curve (AUC) value of the RER curve over entire 2-week BAP exposure between BAP and vehicle fed group (right). **H,** Linear regression plot of body weight percentage and RER AUC in female CD-1 mice. **I,** The effects of BAP on locomotor activity over the 2-week exposure in female. **J, K**, The effects of 13 days of BAP or vehicle emulsion feeding on **(J)** interscapular temperature in female mice with **(K)** representative images. **L-N,** Changes in mRNA expression of markers associated with **(L)** browning, **(M)** catecholamine signaling, and **(N)** fatty acid oxidation in the inguinal WAT (iWAT) of female CD-1 mice after 2 weeks of BAP or vehicle feeding. **O–R,** Changes in **(O)** body weight as a percentage of starting weight, **(P)** fat tissue mass, **(Q)** daily food intake, and **(R)** energy expenditure (EE) in male CD-1 mice fed 300 mg/kg/day BAP or vehicle emulsion for 2 weeks (*n* = 8). **S,** Regression plot of energy expenditure from male mice fed BAP or vehicle emulsion over the second week of the experiment (Day 8–14), *n* = 7–8. **T,** A representative fragment of the effects of BAP on RER in male CD-1 (left). The differences between total AUC value of the RER curve over entire 2-week BAP exposure between BAP and vehicle fed group (right). **U,** The effects of BAP on locomotor activity over the 2-week exposure in male. **V**, The effects of 13 days of BAP or vehicle emulsion feeding on interscapular temperature in the male mice. **W, X,** Changes in mRNA expression of markers associated with **(W)** browning and **(X)** fatty acid oxidation in the epididymal WAT (eWAT) of male CD-1 mice after 2 weeks of BAP feeding. **Y, Z**, A summary figure of **(Y)** the effects of BAP on WAT function markers expression and **(Z)** the overall *in vivo* effects of BAP in female and male CD-1 mice. Data were analyzed using two-way ANOVA with Bonferroni post-hoc test for all daily measurements of body weight percentage and food intake **(B, D, E, O, Q, R).** Unpaired two-tailed *t*-test was used for 2 group comparisons **(C, G, I, J, L-N, P, T-X).** ANCOVA analysis controlled for body weight **(F, S).** Values are presented as mean ± SEM with *P* values. *P*-values greater than 0.2 are presented as ns. Δ represent the net differences in body weight percentage between the BAP group and the vehicle control group.

Obesity can occur with both increased energy intake and decreased energy expenditure, thus improving either is an effective strategy to promote weight loss. Since female mice exhibited significant weight loss with BAP independent of appetite, we questioned whether BAP regulates energy expenditure and thermogenesis. Female and male CD-1 mice were placed on a 60% HFD for 8 and 4 weeks, respectively, followed by 2 weeks of daily BAP feeding (female: 200 mg/kg/day, male: 300 mg/kg/day). One week before BAP exposure, all mice were moved into single-housed metabolic cages for acclimation and were kept in the same cage for the remainder of the experiment to measure energy expenditure (EE), respiratory exchange ratio (RER), and physical activity (Figure 4A). Both inguinal WAT (iWAT) and eWAT were dissected and weighed at the end of the experiment. In females, BAP-fed mice had significantly lower body weight (5.70%) compared with vehicle-fed controls after 2 weeks, consistent with the previous female cohorts (Figure 4B, Fig. S2A). No significant changes in iWAT and eWAT mass were observed (Figure 4C). Similarly, no significant changes in food intake and water intake were detected, further reinforcing an appetite independent mechanism of BAP-mediated weight loss in the females (Figure 4D, Fig. S2B and S2C). To assess whether BAP affects EE in these mice, we performed analysis of covariance (ANCOVA) to compare the differences in average EE between vehicle and BAP-fed mice over the second week of the exposure period (day 8–14), controlling for the effects of body weight on EE (Figure 4E, F). However, perhaps surprisingly, no significant differences were detected between the vehicle and BAP-fed groups (ANCOVA, *P* = 0.62), further implying that BAP does not change EE in these females.

We then examined the effect of BAP on RER, which provides an estimate of substrate utilization preference, with an RER close to 0.7 representing primarily fat/lipid utilization and an RER close to 1.0 indicating primarily carbohydrate/glucose utilization. Female BAP-fed mice displayed significantly lower RER over the 2-week feeding period, indicating a preference for using fat as their primary energy fuel (Figure 4G). Moreover, RER were positively correlated with body weight change of these female mice, implying that changes in RER may be involved in the weight loss effects (Figure 4H). No changes in locomotor or physical activity were detected, indicating mice fed BAP maintained their normal behavior (Figure 4I, Fig. S2D). In addition, a trend towards increased interscapular temperature (*P* = 0.093) was observed in female BAP-fed mice, suggesting potential improvements in thermogenic functions (Figure 4J, K).

To further investigate the underlying mechanisms of enhanced lipid utilization and potentially thermogenesis in the female mice, we measured several markers of browning and lipid metabolism in their iWAT and eWAT. Interestingly, although there were no significant changes in eWAT, BAP significantly upregulated the mRNA expression of multiple thermogenesis-associated genes in the iWAT, including *Ucp1*, elongation of very long chain fatty acids 3 (*Elovl3*), and creatine kinase B (*Ckb*) (Figure 4L, Fig. S2F). Similarly, a significant increase in cytochrome C oxidase subunit 7A (*Cox7a*), a regulator of mitochondrial respiration, was observed only in the iWAT but not eWAT, indicating a depot-specific effect of BAP on modulating WAT browning in females (Fig. S2E and S2H). In rodents and humans, adipose tissue browning is mainly promoted by sympathetic activation via β-adrenergic receptors and catecholamine signaling [[65], [66], [67]]. BAP significantly increased the mRNA expression of β2-adrenergic receptor (*Adrb2*) and tyrosine hydroxylase (*Th*), the rate-limiting enzyme for catecholamine synthesis, in the iWAT of female mice, further implicating its ability to enhance WAT browning (Figure 4M). Concurrently, BAP upregulated the expression of pyruvate dehydrogenase kinase 4 (*Pdk4*), a key mitochondrial enzyme that shifts cellular metabolism from glucose oxidation towards fatty acid oxidation, in the iWAT of female mice (Figure 4N). A trending increase in carnitine palmitoyl transferase 1 b and c (*Cpt1b*: *P* = 0.089, and *Cpt1c*: *P* = 0.062) was also seen in the iWAT, suggesting BAP could also enhance the capacity for β-oxidation (Figure 4N). Like the patterns observed with the browning makers, the induction of these lipid oxidation enzymes primarily occurred in the iWAT, whereas only a slight increase in *Cpt1c* and a trending increase in *Pdk4* was detected in the eWAT (Fig. S2G).

In the male mice, 2 weeks of BAP feeding significantly decreased body weight, iWAT mass, and food intake, aligning with what we observed in our previous male cohorts (Figure 4O-Q, Fig. S2I-S2K). No significant changes in EE were detected (ANCOVA, *P* = 0.86), indicating the weight loss effect of BAP in male mice is mainly caused by appetite suppression (Figure 4R, S). Meanwhile, RER trended towards a decrease in male mice fed BAP but was unable to reach statistical significance (*P* = 0.064) (Figure 4T). No changes in locomotor activity nor interscapular temperature were found in the male mice (Figure 4U, V, Fig. S2L).

Consistent with what we observed in the previous 2- and 4-week male cohorts, BAP significantly increased *Ucp1* mRNA expression in the eWAT, whereas no changes in other markers of browning nor mitochondrial respiration were detected (Figure 4W, Fig. S2M). Interestingly, *Ucp1* induction by BAP in males occurs only in eWAT, but not in iWAT, directly contrasting with the pattern observed in females (Figure 4L, W, Fig. S2F, S2N). Likewise, a trend towards increased *Pdk4* (*P* = 0.067) and a significant decrease in adipogenesis marker peroxisome proliferator-activated receptor gamma (*Pparg*) was only detected in the eWAT of male BAP-fed mice, whereas no significant changes were observed in the iWAT (Figures 4X, Fig. S2O, S2P).

Overall, in comparison, BAP induced greater changes specifically in the iWAT of female mice, including increased markers of browning and lipid oxidation, even at a lower feeding dose (Figure 4Y). Although BAP caused a similar weight-loss phenotype in female and male CD-1 mice, the mechanisms mediating weight loss and its effects on specific metabolic parameters, such as appetite, circulating metabolic hormones, liver health, adipose tissue function, and substrate utilization, appear to be sex-specific (Figure 4Z, Fig. S2Q).

### 2.5 BAP modulates feeding neuropeptide expression in hypothalamic neuronal models

**Figure 5:**
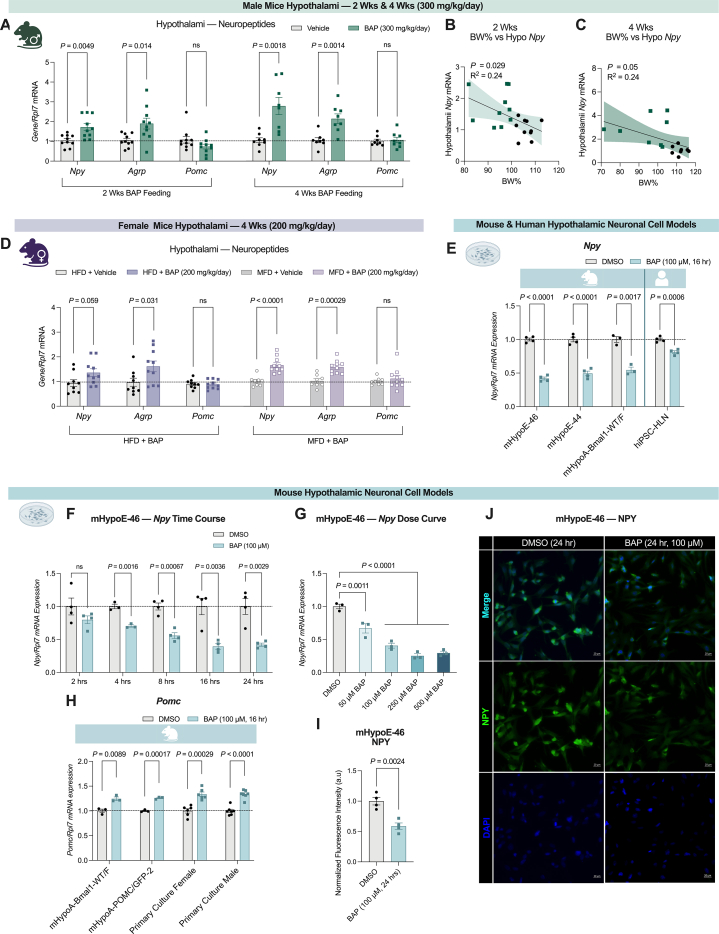
**BAP modulates hypothalamic feeding neuropeptide expression**. **A, D,** The effect of oral BAP feeding on the mRNA expression of hypothalamic feeding neuropeptides, *Npy, Agrp, Pomc*, in the hypothalamus, including the **(A)** male groups that underwent 2 (*n* = 10) or 4 weeks (*n* = 8) of 300 mg/kg/day BAP, and **(D)** female 4 weeks 200 mg/kg/day BAP while on HFD or MFD (*n* = 8–10). **B, C,** Final body weight as a percentage of starting weight was correlated to hypothalami *Npy* mRNA levels of the **(B)** 2-week and **(C)** 4-week male cohorts using simple linear regression with 95% confidence bands around the best-fit line (*n* = 10–8). **E,** Changes in *Npy* mRNA expression in mHypoE-46 (*n* = 4), mHypoE-44 (*n* = 4), mHypoA-Bmal1-WT/F (*n* = 3), and hiPSC-HLN (*n* = 4) treated with 100 μM BAP for 16 h. **F,** Changes in *Npy* expression after 2-to-24-hour treatment with100 μM BAP in mHypoE-46 neuronal cell line (*n* = 4). **G,** Changes in *Npy* expression after treatment of mHypoE-46 (*n* = 3) cell line with increasing concentrations of BAP for 16 h. **H,** Changes in *Pomc* mRNA expression in mHypoA-Bmal1-WT/F (*n* = 3), mHypoA-POMC/GFP1 (*n* = 3), and primary hypothalamic cultures were generated from 8 weeks old male (*n* = 7) and female (*n* = 6) CD-1 mice treated with 100 μM BAP for 16 h. **I, J,** Changes in **(I)** normalized fluorescence intensity of NPY in mHypoE-46 cells treated with 100 μM BAP or DMSO for 24 h with **(J)** representative ICC images. Data was analyzed using unpaired two-tailed *t*-test when comparing neuropeptide expressions between vehicle and BAP groups **(A, D, E, H, I).** Two-way ANOVA with Bonferroni post-hoc test was used for the analysis of the timecourse **(F),** and One-way ANOVA with Bonferroni post-hoc test was used for the dose curve analysis **(G).** Values were normalized to vehicle. Values are presented as mean ± SEM with *P* values. *P*-values greater than 0.2 are presented as ns.

Due to the consistent appetite suppression phenotype observed in the male mice, we first investigated the potential anorexigenic actions of BAP in the hypothalamus, a brain region critical for appetite regulation. Unexpectedly, an increased *Npy* and *Agrp* mRNA expression were observed in the hypothalami of male mice subjected to 2- or 4-week BAP feeding, despite having an anorexigenic phenotype (Figure 5A). The increased hypothalamic *Npy* was also found to be negatively correlated with body weight change upon BAP feeding, leading us to hypothesize that the induction in orexigenic neuropeptides may be a compensatory mechanism by which the mice attempt to regain their body weight (Figure 5B, C) [68]. A similar increase in *Npy* and *Argp* was seen in the female mice with weight loss but no significant changes in food intake, reinforcing the idea of a compensatory mechanism (Figure 5D). Additionally, as these hypothalamii were collected at the end of the experiment (∼18 h after the last dose) rather than a short amount of time from the initial exposure, these neuropeptide changes are more likely to represent a long-term consequence of BAP-mediated weight loss instead of an early and active response to BAP exposure.

Neuropeptide expression *in vivo* is influenced by multiple factors such as body weight, hormonal feedback, and circadian rhythms, which could potentially mask the actions of BAP [69]. The hypothalamus is a complex neuroendocrine region containing multiple cell types and distinct neuron subpopulations that can have divergent responses even to the same stimuli [[69], [70], [71]]. To isolate the direct effects of BAP on neuropeptide expression in individual neuronal subpopulations, multiple *Npy-* and *Pomc*-expressing hypothalamic neuronal cell lines were treated with 100 μM BAP for 16 h. BAP consistently decreased *Npy* mRNA expression in murine-derived *Npy*-expressing hypothalamic neuronal models, with the strongest suppression occurring in the mHypoE-46 (∼60%) and mHypoE-44 (∼50%) cell lines (Figure 5E). Further time-course (2–24 h) experiments conducted in these two cell lines revealed the earliest significant reduction in *Npy* mRNA after 4 h of BAP exposure with the suppression increasing over time until it plateaued at 16 h (Figure 5F, Fig. S3A). Increasing doses of BAP strengthened the downregulation of *Npy*, with a ∼70% decrease occurring with the 250 and 500 μM doses in the mHypoE-46 and mHypoE-44 cells (Figure 5G, Fig. S3B). Considering *Npy* was not substantially suppressed greater beyond the 100 μM dose, it was selected as the optimal dose for our following *in vitro* mechanistic experiments. A significantly decreased NPY fluorescence intensity was observed in the mHypoE-46 cells after 24 h of 100 μM BAP treatment, showing that BAP suppressed NPY protein as well (Figure 5I, J). To assess the translatability of our results in human neuronal models, hypothalamic-like neurons differentiated from human induced pluripotent stem cells (hiPSC-HLN) were treated with 100 μM BAP for 16 h, resulting in a significant decrease in *Npy* mRNA by 19% (Figure 5E). Concurrently, BAP increased *Pomc* expression by ∼25% in *Pomc*-expressing cell lines, mHypoA-BMAL1-WT/F and mHypoA-POMC/GFP-2, as well as primary hypothalamic neurons extracted from 8-week-old male and female CD-1 mice (Figure 5H). Overall, we found that BAP exerts an anorexigenic tone in murine- and human-derived hypothalamic neuronal models by decreasing *Npy* and increasing *Pomc*.

### 2.6 BAP inhibits EGFR/ErbB2, MAPK/ERK and mTORC1 cascades in hypothalamic neuronal cells

Next, we aimed to elucidate the mechanisms by which BAP acts in hypothalamic neurons to exert its anorexigenic function, with the mHypoE-46 cells as our *in vitro* model. The clonal *Npy*-expressing mHypoE-46 line is well characterized: expresses neuron-specific and neurosecretory markers, has intracellular calcium responses after depolarization, and properly responds to hormonal and nutrient stimuli [[72], [73], [74], [75], [76], [77]].

As previously mentioned, there is no homologous receptor system for BAP in mammals [27]. Two prior studies have suggested that BAP could act in mammalian cells by antagonizing P2 purinergic receptors (P2Rs) or activating protein kinase A (PKA) in a cAMP-independent manner [78,79]. Thus, we first examined whether activating P2Rs with endogenous ligands would limit or block the effects of BAP. Co-treatment of mHypoE-46 cells with P2R ligands, ATP, UDP, and UTP, were unable to block the effect of BAP on *Npy* (Fig. S3C–S3E). Furthermore, pharmacological inhibition of PKA with protein kinase inhibitor peptide (PKI) or H89 were unable to block the downregulation of *Npy* by BAP, indicating BAP is unlikely to signal through PKA (Fig. S3F and S3G).

**Figure 6:**
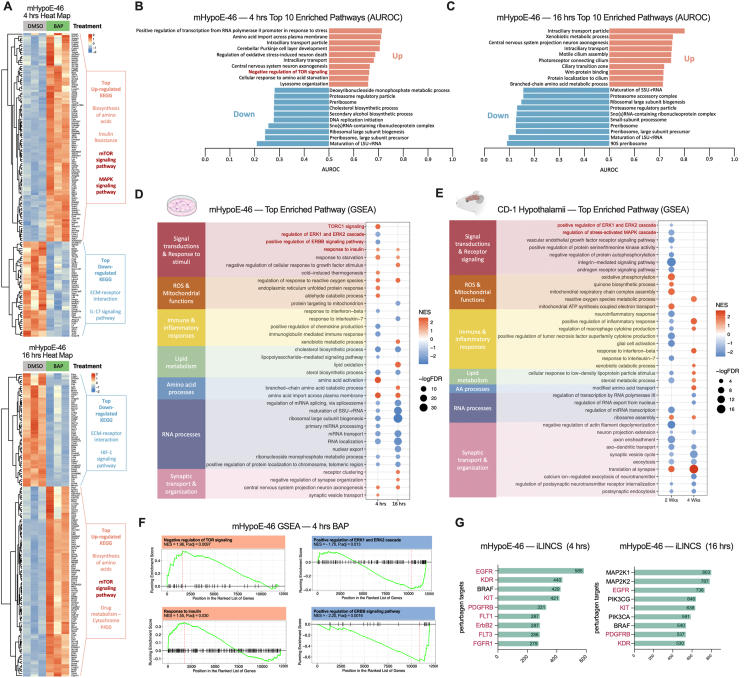
**Bioinformatic analysis of transcriptome changes in BAP-treated mHypoE-46 neurons and hypothalami of BAP-fed CD-1 male mice**. **A,** RNA-seq heatmap of the top 150 DEGs based on padj. value in mHypoE-46 neurons treated with 4 and 16 h of 100 μM BAP (*n* = 3). Over-represented pathways were determined using DAVID ORA analysis and were listed on the right. **B,C,** Top 10 GO pathways up- and down-regulated by **(B)** 4 or **(C)** 16 h of BAP treatment in mHypoE-46 cells, determined by threshold-free AUROC method using ErmineR GO analysis. **d-f,** Top enriched pathways identified by GSEA in **(D)** mHypoE-46 neurons treated with 4 or 16 h of BAP (*n* = 3), and **(E)** whole hypothalami of male CD-1 mice after 2 or 4 weeks of 300 mg/kg/day BAP feeding (*n* = 8). **(F)** Individual enrichment plots of pathways that are changed by BAP at 4 h in mHypoE-46 neurons and are related to receptor/signal transductions. **G,** iLINCS analyses of the top perturbagens targets that are concordant with the transcriptomic signature of BAP-treated (100 μM, 4 or 16 h) mHypoE-46 neurons. Targets that belong to the receptor tyrosine kinases family are shown in pink.

To identify alternative pathways by which BAP may act, we conducted RNA-sequencing (RNA-seq) to measure transcriptomic changes in the mHypoE-46 cells treated with 100 μM BAP for 4 and 16 h, which are the earliest and strongest times of *Npy* suppression, respectively (Figure 5F, Fig. S3H). Pathway enrichment analysis was then conducted using three complementary approaches: (1) over-representation analysis (ORA) of the top differentially expressed genes (DEGs) (Figure 6A), (2) area under the receiver operating characteristic curve (AUROC) that uses the genes ranked by degree and direction of differential expression (Figure 6B, C), and (3) gene set enrichment analysis (GSEA) of the full transcriptome (Figure 6D). Notably, a significant and consistent enrichment of the mTORC1 pathway was detected across all algorithms in BAP-treated cells (Figure 6A,B, D, F, Fig. S3I). Hypothalamic mTORC1 signaling plays a pivotal role in regulating energy homeostasis and neuropeptide expression [[80], [81], [82], [83]]. Subsequent RT-qPCR validation confirmed that BAP increased the mRNA expression of several mTORC1 inhibitors, including *sestrin 2* (*Sesn2*), *DNA damage-inducible transcript 4* (*Ddit4*), and *tuberous sclerosis complex 2* (*Tsc2*), as well as downstream effectors inhibited by mTORC1, such as *unc-51-like kinase 1* (*Ulk1*) and *eukaryotic translation inhibition factor 4E-binding protein 1* (*Eif4ebp1*), suggesting an inhibitory action of BAP on mTORC1 activity (Fig. S4A and S4B). Concurrently, BAP significantly downregulated the mitogen-activated protein kinase (MAPK), extracellular signal-regulated kinase (ERK1/2), and epidermal growth factor receptor (EGFR/ErbB) pathway clusters (Figure 6D,F, Fig. S3J). The EGFR/ErbB receptor family acts upstream of signaling cascades including MAPK/ERK and mTORC1, and its dysfunction has been linked to obesity and associated metabolic disorders [[84], [85], [86], [87]]. BAP robustly decreased the mRNA expression of 5 out of the 7 endogenous EGFR ligands, including *amphiregulin* (*Areg*)*, betacellulin* (*Btc*)*, epiregulin* (*Ereg*)*, transforming growth factor alpha* (*Tgfa*), and *heparin binding-EGF like growth factor* (*Hb-egf*), as well as immediate downstream transcription factors of MAPK/ERK, such as *early growth response proteins 1/2/3* (*Egr1/2/3), cFos,* and *cJun*, indicating a broad inhibitory action of BAP on the EGFR/MAPK/ERK pathway (Fig. S4C–S4E). Similar induction of *SESN2*, *DDIT4*, *EIF4EBP1*, and downregulation of *EGR3* were also observed in human-derived iPSC-HLN by BAP (Fig. S4F). Moreover, we observed consistent upregulation of the “response to insulin” pathway at both time points, which could be due to the suppressive role of BAP on MAPK/ERK and mTORC1, as their overactivation induces insulin resistance (Figure 6F, Fig. S3K) [88].

Next, to determine whether changes in these candidate pathways were restricted to the mHypoE-46 cells, similar RNA-seq and bioinformatic analysis was conducted in the whole hypothalami of male CD-1 mice fed 300 mg/kg/day BAP for 2 or 4 weeks (Fig. S3H). GSEA revealed a similar downregulation of the MAPK and ERK1/2 cascades in the 2-week cohort, showing a congruent inhibitory effect of BAP on hypothalamic MAPK/ERK signaling *in vitro* and *in vivo* (Figure 6E, Fig. S3L, S3M).

In parallel, we performed an integrated Library of Integrated Network-based Cellular Signatures (iLINCS) perturbagen analysis to correlate the transcriptomic signature of BAP-treated mHypoE-46 cells with signatures of other known pharmacological compounds [89]. This approach enables us to identify molecules that cause similar gene expression changes to BAP, thereby providing information about its mechanism of action. Interestingly, several top-ranking molecules that are concordant with BAP are small molecule inhibitors that target EGFR, V-Raf Murine Sarcoma Viral Oncogene Homolog B (BRAF), and MEK1/2, all of which are upstream kinases that can activate ERK1/2 and mTORC1 (Figure 6G). Moreover, most of the top concordant molecules target members of the receptor tyrosine kinase (RTK) family, of which EGFR/ErbB is a member (Figure 6G).

**Figure 7:**
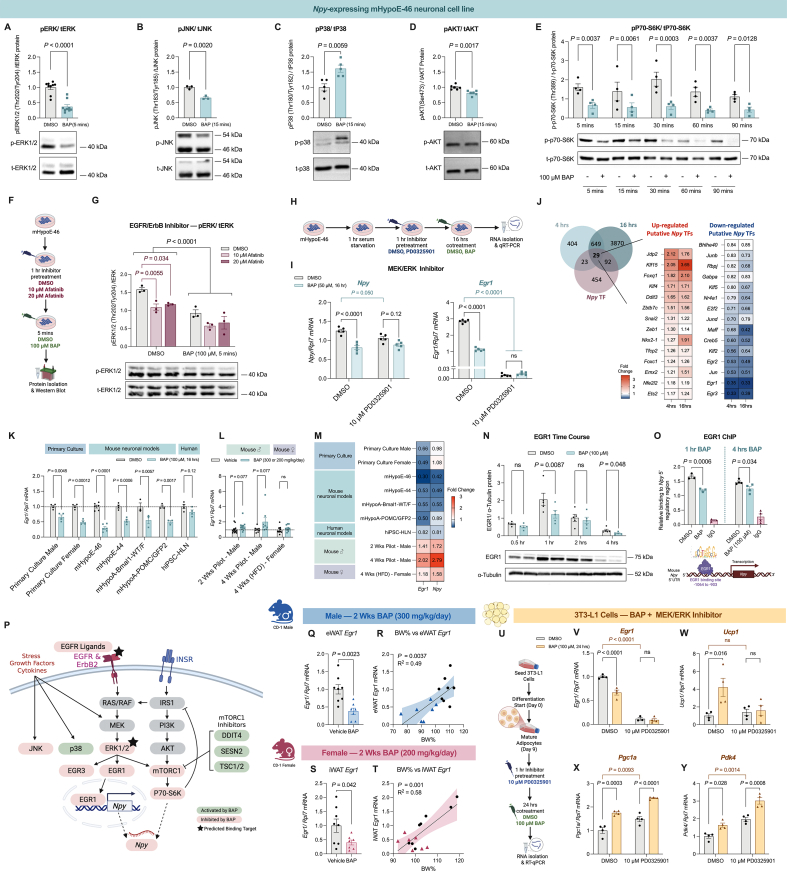
**Delineating the molecular pathways modulated by BAP in hypothalamic neurons and 3T3-L1 cells**. **A-D,** The effect of 5 or 15 min 100 μM BAP treatment on the phosphorylation of **(A)** ERK1/2 (Thr202/Tyr204) (*n* = 9), **(B)** JNK (Thr183/Tyr185) (*n* = 3), **(C)** p38 (Thr180/Tyr182) (*n* = 5), and **(D)** AKT (Ser473) (*n* = 6) in mHypoE-46 cells. **E,** Changes in the phosphorylation levels of p70-S6K (Thr389) in mHypoE-46 neurons treated with 100 μM BAP over a 90 min timeourse (*n* = 4). **F,** Treatment outline for dual EGFR/ErbB2 inhibitor afatinib pretretment experiment. **G,** Changes of ERK1/2 phosphorylation in mHypoE-46 cells pretreated with 10 μM or 20 μM afatinib or DMSO for 1 h, followed by 5 min exposure with 100 μM BAP or DMSO. **H,** Treatment outline for MEK/ERK inhibitor PD0325901 pre/cotreatment experiments. **I,** Changes of *Npy* and *Egr1* mRNA expression in mHypoE-46 neurons pre/cotreated with DMSO or 10 μM PD0325901 for 1 h, followed by 16 h with 50 μM BAP or DMSO. **J,** Venn diagram that cross-references DEGs at 4 and 16 h based on the RNA-seq of BAP-treated mHypoE-46 and a list of putative TFs of *Npy* generated using JASPAR and ENCODE ChIP-seq database. **K,** The effect of 16 h of 100 μM BAP treatment on the mRNA expression of *Egr1* in hypothalamic neuron primary culture from male and female CD-1 mice, and hypothalamic neuronal models from mice and humans (*n* = 3–6). **L,** The effect of BAP feeding on the mRNA expression of *Egr1* in male and female CD-1 mice after 2 or 4 weeks of BAP feeding (300 mg/kg/day for males, 200 mg/kg/day for females) while on HFD (*n* = 8–13). **M,** Heatmap of the fold change of *Egr1* and *Npy* mRNA expression in BAP-treated hypothalamic neuronal models (*n* = 3–6), primary cultures (*n* = 4), and hypothalami of BAP-fed mice (*n* = 8–13), relative to their respective vehicle controls. **N,** Changes in EGR1 protein expression in mHypoE-46 neurons treated with 100 μM BAP in a 4 h timecourse. **O,** ChIP analysis of relative EGR1 or IgG binding to mouse *Npy* 5′UTR after 1 h or 4 h of 100 μM BAP or DMSO treatment in mHypoE-46 neurons (*n* = 3–4). **P.** Summary schematic of the current understanding of the effects of BAP exposure on molecular signal transduction in mHypoE-46 hypothalamic neurons. Green and red represent signaling components that are activated or inhibited by BAP, respectively. Black starts representing putative binding targets of BAP identified by SwissTargetPrediction. **Q-T,** The effects of 2 weeks oral BAP feeding on *Egr1* mRNA expression in the **(Q)** eWAT and **(S)** iWAT of male and female CD-1 mice, respectively (*n* = 8). Body weight change as a percentage of starting weight was correlated to WAT *Egr1* mRNA levels of 2-week BAP and vehicle fed **(R)** male and **(T)** female CD-1 mice using simple linear regression with 95% confidence bands around the best-fit line (*n* = 8). **U,** Differentiation and treatment outline for the 3T3-L1 adipocyte cells. **V–Y,** Changes in mRNA expression of **(V)***Egr1*, **(W)***Ucp1*, **(X)***Pgc1a*, and **(Y)***Pdk4* in 3T3-L1 cells pretreated with 10 μM PD0325901, followed by 24 h cotreatment with 100 μM BAP or DMSO (*n* = 4). Data were analyzed using unpaired two-tailed *t*-test for 2 components comparisons **(A-D, J, L, O, Q, S),** and Two-way ANOVA with Benjamini test was used to analysis with two variables **(E, G, I, N, V–Y)**. One-way ANOVA with Bonferroni post-hoc test was used for ChIP analysis **(O).** Values were normalized to vehicle/DMSO controls. A representative western blot image was included below each western blot analysis. Values are presented as mean ± SEM with *P* values. *P*-values greater than 0.2 are presented as ns.

Next, we wanted to validate the effect of BAP on the signal transduction of MAPK and mTORC1 *in vitro*. The mammalian MAPK family of kinases contains three subfamilies: ERK, c-Jun N-terminal kinases (JNKs), and p38 mitogen-activated protein kinases (p38s). Acute 100 μM BAP exposure robustly decreased the phosphorylation of ERK1/2 (Thr202/Tyr204) and JNK (Thr183/Tyr185) at 5 and 15 min, respectively, but increased p38 phosphorylation at Thr180/Tyr182, demonstrating a selective targeting of MAPK components by BAP (Figure 7A–C). In addition, we assessed the phosphorylation of mTORC1 upstream activator AKT and downstream effector p70-S6K. BAP modestly decreased AKT phosphorylation (Ser473) after 15 min and robustly suppressed p70-S6K phosphorylation (Thr389) over a 5 to 90-minute time course (Figure 7D, E). Thus, BAP-mediated inhibition of mTORC1/S6K is likely caused by direct targeting of mTORC1 or ERK1/2, with minimal contribution from AKT. In addition, since our iLINCS analysis showed BAP concordant with multiple RTK inhibitors, we examined whether BAP acts as a broad RTK inhibitor by performing a mouse phospho-RTK array (Fig. S5A). Acute 5-minute BAP exposure to the mHypoE-46 cells specifically suppressed EGFR and ErbB2 tyrosine phosphorylation with no impact on other RTKs tested (Fig. S5B and S5C).

### 2.7 ERK1/2 inhibition by BAP does not depend on EGFR and ErbB2

As previously mentioned, no validated mammalian binding partners of BAP have been described in the literature. Thus, to identify the putative binding targets of BAP, we used SwissTargetPrediction, an algorithm that predicts macromolecular targets of small molecules [90]. Of note, EGFR and ERK2 were identified as top binding candidates for BAP in rodents, which was consistent with our prior pathway analysis (Fig. S5D). Hypothalamic EGFR and MAPK/ERK signaling are involved in the regulation of *Npy* in response to hormones and external stimuli [[91], [92], [93]]. Treatment with EGF, an endogenous EGFR ligand, can induce *Npy* expression in PC-12 rat pheochromocytoma cells [94] and mHypoE-46 cells (Fig. S5E). Since EGFR and ErbB2 are common receptors upstream of ERK1/2, we first wanted to determine whether BAP-mediated ERK inhibition depends on upstream EGFR/ErbB2. We pre-treated the mHypoE-46 cells with 10- or 20-μM afatinib, an irreversible dual EGFR/ErbB2 inhibitor, for 1 h, followed by 5 min of exposure to 100 μM BAP or vehicle (Figure 7F). Afatinib pre-treatment significantly decreased ERK1/2 phosphorylation in the mHypoE-46 cells, but BAP was able to suppress it further, suggesting that BAP is likely targeting ERK directly to inhibit its activity, independent of upstream receptors (Figure 7G).

### 2.8 BAP-mediated *Npy* downregulation depends on MEK/ERK/EGR1 inhibition

To determine the involvement of EGFR and ERK in the *in vitro* effects of BAP, the mHypoE-46 cells were pretreated with selective inhibitors for EGFR (gefitinib, 5 μM) or MEK/ERK (PD0325901, 10 μM), followed by BAP or DMSO cotreatment (Figure 7H). EGFR inhibition via gefitinib did not change *Npy* expression nor did it prevent *Npy* suppression by BAP (Fig. S5F). Meanwhile, MEK/ERK inhibition using PD0325901 significantly decreased *Npy* expression by 18% but was unable to block the effect of 100 μM BAP on *Npy* (Fig. S5G). Besides *Npy* downregulation, PD0325901 treatment itself caused similar gene expression changes as BAP in the cells, such as decreasing *Egr1*, *Egr3*, and increasing *Sesn2*, suggesting it may have a similar molecular mechanism as BAP (Fig. S5H–S5K). Notably, when co-treating with 50 μM BAP, PD0325901 successfully blocked *Npy* downregulation by BAP and completely abolished the effect of BAP on decreasing *Egr1*, *Egr3*, *Hb-egf*, *Areg*, *Ereg*, and upregulating *Sesn2* (Figures 7I, Fig. S5L-S5P). These results indicates that MEK/ERK inhibition is a contributing pathway by which BAP acts *in vitro* to exert its actions. However, at higher concentrations, BAP may modulate multiple pathways simultaneously to achieve its full efficacy.

Lastly, to ascertain the connection between BAP-mediated ERK inhibition and *Npy* mRNA downregulation, we investigated putative transcription factors (TFs) of the *Npy* gene that are downstream of ERK. To do so, we cross-referenced the RNA-seq data with a list of putative *Npy* TFs generated using JASPAR and ENCODE ChIP-seq databases [95]. From this, 29 putative TFs of *Npy* were identified to be significantly changed by BAP at 4 and 16 h (Figure 7J). Notably, this included *Egr1,* robustly decreased by BAP, and whose synthesis depends on MEK/ERK activation [96]. Moreover, previous ChIP-seq analysis from Natt et al. revealed an enriched EGR1 binding site in the 5′ upstream regulatory region of *Npy* in the mouse dorso/ventrolateral striatum, indicating that EGR1 is associated with *Npy* transcription in the brain [97]. To validate EGR1 as a target of BAP, we surveyed multiple murine and human neuronal models and found that BAP consistently and robustly decreased *Egr1* expression in a pattern that correlates with *Npy* (Figure 7K, M). Conversely, in the whole hypothalamus of male and female CD-1 mice where *Npy* increased with BAP-feeding, *Egr1* was also increased by BAP (Figure 7L, M). Subsequent western blotting and ChIP revealed significantly lower EGR1 protein expression, along with reduced EGR1 binding to the 5′ upstream regulatory region of the mouse *Npy* gene after 1- and 4-hour of 100 μM BAP treatment, confirming the transcriptional regulatory role of EGR1 on *Npy* by BAP (Figure 7N, O). Overall, our results suggested that BAP regulates *Npy* expression in *Npy*-expressing hypothalamic neuronal cells primarily through MEK/ERK inhibition, which subsequently decreased EGR1 mRNA and protein levels, thereby reducing its binding to the *Npy* 5′ regulatory region (Figure 7P).

### 2.9 MEK/ERK/EGR1 inhibition is involved in BAP-mediated changes in white adipocytes

We questioned whether the MEK/ERK/EGR1 pathway is also involved in the actions of BAP in other cell types. Oral BAP feeding altered white adipose tissue function *in vivo*, such as promoting browning and enhancing fatty acid oxidation. Lower EGR1 levels in WAT have several metabolic benefits, including lower body weight, enhanced WAT browning, and improved insulin sensitivity in rodents [[98], [99], [100]]. In humans, *EGR1* mRNA expression in the subcutaneous WAT is positively associated with BMI and insulin resistance [99,101]. Notably, in our animal experiments, BAP significantly decreased *Egr1* mRNA expression in the iWAT and eWAT of females and in the eWAT of male CD-1 mice (Figure 7Q,S, Fig. S6A-S6D). More importantly, WAT *Egr1* expression was strongly and positively correlated with body weight change, as mice with greater weight loss after BAP feeding had lower *Egr1* expression in their WAT (Figure 7R, T, Fig. S6D). To assess whether WAT *Egr1* downregulation by BAP also depends on MEK/ERK inhibition, we treated 3T3-L1 derived adipocyte cells with a similar PD0325901 pre/co-treatment paradigm as previously performed (Figure 7U). Treatment with PD0325901 for 24 h alone significantly decreased *Egr1* mRNA expression in the 3T3-L1 cells and completely abolished the effect of BAP on *Egr1* downregulation (Figure 7V). Furthermore, BAP significantly increased thermogenic markers *Ucp1* and *Pgc1a*, as well as lipid oxidation marker *Pdk4*, after 24 h, consistent to the phenotype observed *in vivo* (Figure 7W-Y). Pre/co-treatment with PD0325901 blocked BAP-mediated *Ucp1* induction, while the inhibitor itself has no effects on *Ucp1* expression. Conversely, MEK/ERK inhibition via PD0325901 significantly increased *Pgc1a* and *Pdk4* on its own, but BAP was able to further increase their expression when co-treated. Overall, these results suggested that MEK/ERK/EGR1 inhibition may play a complementary role in the effects of BAP in adipocytes, including the increase of *Ucp1*.

## 3 Discussion

Herein, we showed that the plant hormone BAP represents a promising therapeutic to combat obesity. In our *in vivo* experiments, BAP improved several metabolic parameters associated with obesity, including body weight, fasting blood glucose, hyperinsulinemia, hyperleptinemia, lipid utilization, WAT function, and hepatic MASLD markers, which may be consequences of the weight loss effect. In the male mice, appetite suppression occurs rapidly after BAP feeding, which is likely the primary cause of weight loss, as EE remained unchanged. In addition, as no changes in locomotor and physical activity were observed in BAP-fed mice, the weight loss was not accompanied by significant side effects, such as lethargy or abnormal behaviors. Female BAP-fed mice exhibited stronger weight loss (∼7%) than male mice (∼5%) while receiving a lower dose, aligning with the EPA report, where they mentioned that the female rats were more sensitive to BAP [32]. Although the weight loss outcome is consistent between both sexes, the *in vivo* mechanisms mediating weight loss are sex specific. While the reduced body weight by BAP in males is attributed to appetite suppression, in female mice, BAP-mediated weight loss is independent of food intake and EE, as changes in both were unable to reach statistical significance. Although a slight trending decrease in food intake (*P* = 0.096) was observed in the female metabolic cage cohort, we think this mild and sub-significant change in feeding is not sufficient to explain the magnitude of weight loss of female BAP-fed mice, especially when comparing to the males with robust appetite suppression and similar weight loss. Thus, we think alternative, appetite-independent factors are likely involved in the weight loss mechanisms of BAP in females. Indeed, female mice fed BAP displayed enhanced preference for fat utilization, stronger WAT remodeling including increased browning and lipid oxidation, as well as a trend towards increased interscapular temperature, all of which were not observed in male BAP-fed mice besides *Ucp1* induction in eWAT. The observed weight loss in female BAP-fed mice thus may be caused by a synergistic effect of all these factors, along with a minor contribution from the subtle appetite changes. Future single housing, long-term experiments should be conducted to conclusively determine the effects of BAP on food intake in female mice.

The pathogenic cause and complications of obesity, such as hyperphagia, insulin resistance, neuroinflammation, and adipose tissue remodeling, are known to be sex specific in rodents and humans [[102], [103], [104], [105]]. Female mice are less prone to develop hyperphagia when challenged with a HFD, as they are more leptin-sensitive and have higher hypothalamic *Pomc* mRNA expression during fasting [102]. Moreover, male mice develop rapid astrogliosis and neuroinflammation in the hypothalamus within days of HFD, whereas the females show delayed or milder gliosis and resistance to hyperphagia, potentially through the anti-inflammatory effects of estrogens and preserved leptin sensitivity [[106], [107], [108]]. Thus, the absence of appetite suppression in female BAP-fed mice may be due to the lack of HFD-induced hyperphagia in the first place, making BAP less robust in altering food intake in females compared to males. This possibility could be tested by including a parallel chow-fed control group in future experiments. Alternatively, the weight loss effect of BAP in females may also be caused by decreased lipid or nutrient absorption, which can be tested via measuring fecal lipid content and intestinal lipid absorption rate.

Another sex-specific effect of BAP was significantly higher *Ucp1* expression only occurred in the iWAT of female BAP-fed mice versus in the eWAT of males. Furthermore, changes in WAT function, such as increased browning and lipid oxidation markers, were only observed in the iWAT of the females but not in the males. These discrepancies could be attributed to the sex differences in adipose tissue biology, as the subcutaneous WAT of females tends to have higher expression of mitochondrial genes, thermogenic markers, and greater browning capacity compared to males [104,105,[109], [110], [111]]. Conversely, the eWAT is more metabolically active and critically involved in inflammation and insulin resistance caused by obesity and HFD in male mice [103]. No significant changes in EE were detected in both female and male by BAP when testing at the standard animal facility temperature (22 °C). However, variabilities in basal EE may cause difficulties in observing subtle changes; thus, future studies at thermoneutrality should be conducted.

We recognize that weight loss and lower dietary fat consumption could induce consequential changes in peripheral organs by diminishing ectopic fat accumulation and inflammation. Thus, the observed metabolic benefits in BAP-fed mice, such as WAT browning and reduced hepatic MASLD markers, could be downstream or because of weight loss and appetite suppression, increasing fat metabolism for energy fuel and heat production. However, since female mice displayed greater changes in WAT browning, RER, and interscapular temperature, without significant changes in feeding, we think BAP is likely stimulating WAT browning via its direct actions rather than as a sole consequence of reduced HFD intake. Furthermore, as shown in our *in vitro* experiment using 3T3-L1 derived adipocytes, BAP significantly increased the mRNA expression of *Ucp1* and *Pgc1a* in the absence of external influences, further suggesting that BAP can directly modulate WAT browning markers independent of *in vivo* feedback.

In the whole hypothalamus of male CD-1 mice, BAP increased the expression of orexigenic neuropeptides *Npy* and *Agrp*, contradicting the anorexigenic phenotype we observed in these mice. Similar induction in *Npy* and *Agrp* was also observed in the hypothalamus of female BAP-fed mice without significant changes in food intake. Thus, we think these neuropeptide changes are likely a compensatory mechanism by which the mice are trying to regain body weight rather than an active, immediate response to BAP. Fasting, calorie restriction, and diet induced weight loss, elevate the mRNA expression of *Npy* and *Agrp* while decreasing *Pomc* in rodents [68,112,113]. Rapid weight loss through long-term diet restriction induces hyperphagia during *ad libitum* re-feeding in mice via modulating feeding neuropeptides [114]. Acute feeding experiments could potentially circumvent the compensatory mechanisms and confounding variables caused by weight loss. However, since the pharmacokinetics of BAP in mammalian systems are still not well studied, it is challenging to estimate the precise time point by which BAP induces changes in the hypothalamus. Therefore, to isolate the direct effects of BAP on hypothalamic neurons, we used primary hypothalamic neuron cultures, clonal hypothalamic neuronal cell lines, and human-derived iPSC hypothalamic neuronal models to provide evidence for an overall anorexigenic effect of BAP on feeding neuropeptides in the hypothalamus. Future studies using short-term central delivery of BAP along with single cell approaches could aid with delineating the direct effects of BAP in the hypothalamus *in vivo*. We recognize that BAP may act on other regions of the neuroendocrine apparatus, such as the NTS and PBN of the brainstem, to suppress appetite, independent or in conjunction with the hypothalamus. This possibility is important for future assessment via brain immunohistochemical staining upon BAP exposure and radiolabel BAP tracing to identify the main sites of action.

Bioinformatic analyses and validation demonstrated that BAP selectively modulates MAPK activity by inhibiting ERK1/2 and JNK, and activating p38 in the mHypoE-46 cells, aligning with a previous study that showed differential regulation of MAPK components in human umbilical vein endothelial cells treated with BAP [33]. BAP-induced *Npy* downregulation is mediated by reduced binding of EGR1, an ERK dependent TF, to the 5′ regulatory region of *Npy*. ERK inhibition has been linked to hypothalamic *Npy* regulation in response to insulin and kisspeptin in previous literature [115,116]. Moreover, mice lacking ERK1 or EGR1 or orally fed MEK/ERK inhibitors are resistant to HFD-induced obesity and protected against obesity-associated pathologies, including hyperinsulinemia, insulin resistance and MASLD, exhibiting a similar metabolic phenotype to what we observed in BAP-fed mice [117,118]. Central EGR1 transcriptionally activates *Npy* expression in human SH-SY5Y neuroblastoma cells and the mouse hypothalamus, suggesting BAP may exert its repressive effect through disrupting EGR1 [97,119]. In the periphery, lower EGR1 is associated with improved metabolic health, such as enhanced WAT browning and insulin sensitivity, in animals including humans [[98], [99], [100], [101]]. BAP significantly decreased *Egr1* mRNA expression and increased *Ucp1* in the WAT of obese female and male mice, and in 3T3-L1 cells via MEK/ERK inhibition. Thus, the MEK/ERK/EGR1 pathway is involved in the actions of BAP across different cell types and tissues. In addition, as treatment with PKA inhibitors was unable to block the action of BAP on *Npy* in the mHypoE-46 cells, BAP likely acts through pathways independent of PKA/cAMP. Since the GLP-1R is a G protein-coupled receptor that signals through adenylyl cyclase and subsequently PKA/cAMP [120], BAP may act in parallel with GLP-1R agonists or serve as a secondary option to those who cannot tolerate the current therapies.

Whether BAP is metabolized is an important consideration, since its metabolites may contribute to its weight loss effects. Previous toxicology analyses by the EPA, radiolabelling BAP as benzyladenine-8-C^14^, revealed that BAP was primarily excreted in its intact form in urine within 24 h after consumption in beagle dogs [121]. Another radiolabelling experiment with benzyl-1-C^14^-adenine indicated that all radioactivity was excreted in the urine with most being the parent compound, suggesting that BAP is unlikely to be broken down and accumulate in tissues [121]. For further evidence, our treatment of the mHypoE-46 cells with benzoic acid or adenine, which are possible metabolites of BAP, were unable to recapitulate the effects of BAP on *Npy, Egr1,* and *Sesn2* (Fig. S6E and S6F). Taken together, these results infer that the effects we have observed are caused by intact BAP.

Overall, our preclinical data in diet-induced obese mice and mammalian cell models describe the anti-obesity efficacy of the plant hormone BAP to regulate energy homeostasis via interacting with mammalian signal transduction pathways, such as MEK/ERK. The ability of BAP to suppress appetite and modulate adipose tissue function results in significant weight loss and improvements in obesity related metabolic parameters *in vivo*, demonstrating its translational potential for treating obesity. BAP thus holds promise to serve as a novel and alternative anti-obesity agent for weight loss and maintenance.

## 4 Methods

### 4.1 Animals

For the animal experiments, 7-week-old male and female CD-1 mice were purchased from Charles River Laboratories (Strain code: 482) and were acclimatized to our facilities (Biological Sciences Facility and Division of Comparative Medicine, University of Toronto) 1 week prior to the commencement of our experiments. Mice were housed in pairs in standard caging with Pure-o’Cel bedding and were kept on 12:12 light–dark schedule (7 am-7 pm). All experiments were conducted in accordance with the Canadian Council on Animal Care and approved by the University of Toronto Animal Care Committee (protocol 20012689). Male and female CD-1 mice were fed a diet containing 60% kilocalories from fat (HFD) (Research Diets, D12492) for 4 and 16 weeks, respectively, to induce weight gain and an obese phenotype. During this period, the mice were weighed weekly to track their weight gain. After 3 and 15 weeks respectively, the mice were acclimated to the emulsion feeding protocol over a 1-week period, including 4 days of daily handling acclimation and 3 days of handling with emulsion feeding acclimation. The feeding method was adopted from Kuster et al. and the volume was increased to 200 μL [56]. After the acclimation period, the mice are weight matched and randomly assigned to vehicle (50% honey and 1% carboxymethyl cellulose (CMC)) or BAP (75, 150, 200, or 300 mg/kg BAP in 50% honey and 1% CMC) groups and received their respective emulsion feeding 2 h prior to light off, for 2 or 4 weeks. For the female CD-1 mice experiment, a subset of the mice was randomly assigned to 30% kilocalories from fat diet (MFD) (Research Diets, D20072301) for one week prior to the emulsion acclimation and continued this diet throughout the BAP exposure period. During the exposure period, the mice had their body weight, food intake, and water intake measured daily.

### 4.2 Food intake assessment

All mice were provided with *ad libitum* access to HFD and water. The food pellets were placed in a glass petri dish and placed on the cage floor. The weight of food for each cage was measured daily by manually collecting all pellets and weighing them on a standard laboratory scale. The difference in food weight between each day was recorded as food intake. For cohorts that were double-caged (e.g., 2- and 4-week male cohorts, the male dose curve cohorts, and the female HFD/MFD cohorts), food and water intake data were presented as total intake per cage (i.e., combined intake of 2 mice). For the single-caged cohorts (e.g., the female and male metabolic cage cohorts), food and water intake data were presented as individual intake per mouse.

### 4.3 Indirect calorimetry

Male and female CD-1 mice (7-week-old) were acclimated to our facility (Division of Comparative Medicine, University of Toronto) for 1 week and subsequently fed a 60% HFD for 4 and 8 weeks, respectively, as described above. After 3 and 7 weeks of HFD, respectively, the mice were moved into single-housed Promethion metabolic cages (Sable Systems International, Las Vegas, NV) located in a temperature-controlled cabinet (14:10 light–dark schedule) within the same facility for cage acclimation. All mice we then acclimated to the emulsion feeding protocol for 3 days, followed by 2 weeks of daily BAP feeding within the same metabolic cage with *ad libitum* access to HFD and water. Volume of O_2_ consumed and CO_2_ produced, physical activity, and water consumption were recorded by the metabolic cage system. Food intake was measured daily by manual weighing. At day 13, interscapular temperature was measured using the C5 compact thermal camera (FLIR 89401-0202) at the standard animal facility temperature of 22 C. Three images were taken per each animal, and the average were used to represent the interscapular temperature of each mouse for statistical calculation. Differences in EE between groups were calculated via analysis of covariance (ANCOVA) using R and CalR to control the effect of body weight on EE.

### 4.4 ipGTT and luminex serum hormone analysis

For the 2- and 4-week male cohorts, a subset of mice was randomly selected to undergo an intraperitoneal glucose tolerance test (ipGTT) at the end of the exposure period. The remaining mice were euthanized (cardiac puncture and cervical dislocation), tissues were collected, immediately frozen on dry ice, and stored at −80 °C until processed for analysis. For serum hormone analysis, blood was collected via cardiac puncture and stored in uncoated centrifuge tubes containing protease inhibitor cocktail (Sigma Aldrich, P2714) supplemented with AEBSF (Sigma Aldrich, 101500), and DPP IV inhibitor (Sigma–Aldrich, DPP4-M). The blood was left to clot at room temperature for 1 h, then centrifuged for 10 min at 1,500 g and 4 °C, the supernatant was collected and stored at −80 °C. Serum hormone levels were measured using the mouse metabolic hormone array (Millipore Sigma, MMHE-44K). The assay was conducted at SPARC BioCentre, Hospital for Sick Children, Toronto, Canada. The assay was performed in duplicate on serum samples.

On the day of the ipGTT, the mice were fasted for a total of 6 h prior to the ipGTT and were weighed after 5 h of fasting. The appropriate volume of 20% glucose solution was aliquoted to achieve a dose of 2 g/kg in the mice. The mice were then intraperitoneally injected with glucose solution. Blood glucose was measured immediately prior to glucose injection and 15, 30, 60, 90, and 120 min after injection.

### 4.5 Reagent preparation

To prepare BAP feeding emulsions for the animal experiments, 2% CMC solution was made by rehydrating sodium CMC (Sigma–Aldrich, C4888) with the appropriate amount of distilled water. The amount of BAP (75, 150, 200, or 300 mg/kg) was aliquoted for each mouse and then suspended in the 100 μL of the viscous 2% CMC solution. The suspended BAP was then mixed with 100 μL of 100% Ontario Bee Honey (Farm Boy) to achieve the final emulsion. The vehicle solution was prepared in the same manner with the exclusion of BAP.

For *in vitro* experiments, BAP (Sigma–Aldrich, B3408) was dissolved in anhydrous dimethyl sulfoxide (DMSO) (Sigma–Aldrich, D2650) to reach the desired stock concentration (25–500 mM). The stocks were then diluted 1:1000 in the growth medium.

For the inhibitor pre/cotreatment experiments, 10 mM afatinib (dual EGFR/ErbB2 inhibitor) (Sigma–Aldrich, SML3109), 5 mM gefitinib (EGFR inhibitor) (Tocris Bioscience, 3000/10) and 10 mM PD0325901 (MEK/ERK inhibitor) (New England Biolabs, 79241S) were dissolved in DMSO and stored at −20 °C, then dissolved in 1:1000 in growth media to a final concentration of 20 μM, 5 μM for and 10 μM, respectively. Cells were treated with 1 h with 2.5 mL of inhibitor media, followed by 0.5 mL of media with BAP added to a final concentration of 50 μM or 100 μM for 16 or 24 h.

For the P2 receptor agonist experiments, UDP (Sigma–Aldrich, 94330) was prepared by dissolving in water to prepare 100 mM stock solution and 100 mM ATP (ThermoFisher, R0441) and 100 mM UTP (ThermoFisher, R0471) solutions were obtained from ThermoFisher. The solutions were then diluted 1:1000 in growth medium to achieve treatment doses of 100 μM.

### 4.6 Cell culture

Hypothalamic neurons from adult or embryonic mice were previously immortalized to generate embryonic male derived (mHypoE-46, mHypoE-44), adult female derived (mHypoA-Bmal1/WTF), and adult male derived (mHypoA-POMC/GFP2) neuropeptide expressing cell models that have been characterized [71]. All mouse derived cell lines were cultured in with Dulbecco’s Modified Eagle Medium (DMEM; Sigma–Aldrich, D6046), 5% fetal bovine serum (FBS; Gibco), 1% penicillin/streptomycin (P/S; Gibco, 15140122), 1000 mg/L glucose supplemented with 1X GlutaMAX (Gibco, 12483020).

In the primary culture experiments, neurons from the hypothalami of six 8-week-old male and eight 8-week-old female CD-1 mice (Charles River, Strain code: 482), euthanized via CO_2_ chamber and cervical dislocation, were dispersed onto 60 mm tissue culture dishes (one hypothalamus split across 2 dishes) coated with poly-L lysine (Sigma–Aldrich, P4707). The cells were cultured in Neurobasal-A medium (Gibco, 10888022) supplemented with 10% FBS, 5% horse serum (Gibco, 10888022), 1% P/S, 1 × B27 supplement (Gibco, 17504044), and 1 × GlutaMAX supplement (Gibco, 35050061). After approximately 9 days of growth, the growth media was replaced with treatment media (as described below). Primary culture procedures were conducted in accordance with the regulations of the Canadian Council on Animal Care and approved by the University of Toronto Animal Care Committee.

For hiPSC-HLN experiments, the BJ-iPSC cell line was cultured on Geltrex-coated (1:200 dilution) (ThermoFisher, A1413301) 6-well cell culture plate in SremMAC iPS-Brew XF (Miltenyi Biotech, 130-104-368) containing ROCK inhibitor Y27632 (Cayman Chemicals) until 100% confluent. Cells were then differentiated into hypothalamic-like neurons according to previously established differentiation protocols [122,123]. For the first 48 h (day 0 to day 2), cells were differentiated to neuroectoderm cells via dual SMAD inhibition (1 μM LDN193189 and 10 μM SB431542). From day 2 to day 9, Sonic hedgehog activation (1 μM smoothened agonist and 1 μM purmorphamine) and Wnt inhibition (10 μM IWR1-endo) was used to drive the cells into ventral diencephalon forebrain cells. The cells were then kept in this state and expanded using 10 μM DAPT (gamma secretase inhibitor and 10 nM retinoic acid from day 9 to day 16). At day 13, the cells were split onto new Geltrex-coated 6-well dishes with differentiation media containing retinoic acid. After day 16, 10 ng/mL BDNF was added to induce cell maturation into hypothalamic-like neurons. The cells were maintained with regular media changes (every 3 days) until they reached maturity at day 21 and were treated with 100 uM BAP or DMSO for 16 h.

3T3-L1 preadipocytes (ATCC CL-173) were maintained in DMEM (Gibco, 11965092) with 10% bovine calf serum (Corning, 35-053-CM). For differentiation, cells were seeded in 12-well plates and grown to confluency (day 0). Adipocyte differentiation was induced in DMEM with 10% FBS (Corning, 35-077-CV) using 500 μM 3-isobutyl-1-methylxanthine (IBMX; Sigma, I5879), 5 μg/ml insulin (Sigma, I0516), 1 μM dexamethasone (Sigma, D4902), and 2 μM rosiglitazone (Cayman, 71740), changed every 2 days. On day 4 cells were fed fresh DMEM with 10% FBS and 5 μg/ml insulin every 2 days until treatment. On day 9, matured 3T3-L1 adipocytes were pre-treated with 10 μM PD0325901 (MEK/ERK inhibitor) for 1 h, followed by 24 h of 100 μM BAP or DMSO co-treatment, as described above.

All cells were maintained in 5% CO_2_ at 37 °C. For the immortalized cell lines, cells were split into 60 mm tissue culture plates approximately 24 h before treatment at a 70–80% confluency. Growth media was replaced with treatment media on the day of treatment as described below.

### 4.7 RNA isolation and RT-qPCR

For the mouse hypothalamic tissue, the hypothalami were titrated by passing through a P1000 pipette 5 times followed by a 23-gauge needle 5 times. Total RNA was isolated from the homogenate using the miRVANA PARIS kit (Invitrogen, AM1556). For the white and brown adipose tissue, a 20 mg piece of tissue was dissociated using a tissue homogenizer for 30 s and total RNA was isolated from the homogenate using the fatty tissue RNA isolation kit (Norgen Biotek, 36200). For the liver and kidney tissue, a 30–40 mg section of the tissue was homogenized in Purelink lysis buffer for 30 s before isolation using Purelink RNA isolation mini kit. For the cell line and primary culture experiments, total RNA was isolated using the Purelink RNA isolation mini kit (Invitrogen, 12183025) according to the manufacturer’s instructions with the on-column DNase step (Invitrogen, 12185010) to remove genomic DNA. Regardless of isolation method, RNA quantity and quality were analyzed using the Nanodrop 2000 (ThermoFisher) and 300–1000 ng of complimentary DNA (cDNA) was reverse transcribed using the high-capacity cDNA reverse transcription kit (Applied Biosystems, 4368814). Quantitative reverse transcriptase polymerase chain reaction (RT-qPCR) was performed with 12.5 ng of cDNA, gene-specific primers (Supplemental Table 1), and PowerTrack SYBR green master mix (ThermoFisher, A46112) on a QuantStudio 5 using the manufacturer’s standard cycling instructions. Primer sequence for *Ucp1, Adrb1, Adrb2, Th* were previously published [124]. Data were analyzed with the ΔΔCT method and normalized to a reference gene, 60s ribosomal protein L7 (*Rpl7*) or Histone 3a (*His3a*).

### 4.8 RNA-sequencing and bioinformatics

For the mHypoE-46 RNA-sequencing (RNA-seq) experiment, total RNA samples were isolated from mHypoE-46 cells treated with 100 μM BAP for 4 and 16 h as described above. Total RNA, 1000 ng in 29 μL, was then sent to The Centre for Applied Genomics at the Hospital for Sick Children (TCAG, Toronto) for RNA-sequencing. Library preparation was performed using the NEB Ultra II Directional poly(A) mRNA kit and the samples were run on the NovaSeq flowcell to achieve 30 million paired end reads per a sample.

For the mouse hypothalamus RNA-seq experiment, the whole hypothalamus was collected from male CD-1 mice fed with 300 mg/kg/day BAP or vehicle for 2 or 4 weeks as previously described. RNA samples were isolated as described above and were additionally cleaned using the RNA clean and concentration kit (Norgen Biotek, 23600). Total RNA, 600 ng in 30 μL, was sent to TCAG for RNA-sequencing. The library preparation was performed using the NEB Ultra II Directional poly(A) mRNA kit and the samples were run on the Illumina NovaSeq 10B flowcell to achieve 40 million paired end reads per a sample.

The reads from both runs were then aligned to the mouse genome (GRCm39, M27) using STAR aligner, v.2.6.0c and raw read counts were obtained using htseq-count v.0.6.1p2. Differential gene expression analysis was performed in R v.3.6.1 using DESeq2.

Bioinformatic gene ontology (GO) analysis was conducted using three methods, gene set enrichment analysis (GSEA) of the full transcriptome ranked by Wald statistic using clusterProfiler R package [125], hypergeometric over-representation analysis (ORA) of the top 100 up- and down-regulated genes with DAVID [126], and an non-parametric AUROC approach that uses the genes ranked by differential expression statistic (stat) values to determine enrichment using the ermineJ package [127]. The perturbagens analysis was conducted using the iLINCS web-based platform (http://ilincs.org) to correlate transcriptome signature of BAP-treated mHypoE-46 cells with signatures of chemical perturbagens generated based on the Broad L1000 assay data. Briefly, perturbations concordance with BAP are compounds that are identified to induce similar gene expression changes as BAP by the algorithm [89]. SwissTargetPrediction algorithm was used to predict putative binding targets of BAP based on its molecular structure [90].

### 4.9 Protein extraction and western blotting

For assessing the phosphorylation levels of signal transduction components, mHypoE-46 cells were grown in DMEM supplemented with 5% FBS and 1% P/S to 85–100% confluency. The cells were then serum starved in plain DMEM for 1 h and treated with 100 μM BAP or 0.1% DMSO dissolved prepared in plain DMEM for 5, 15, 30, 60, or 90 min. For the time course to assess EGR1 protein expression, mHypoE-46 cells were treated with 100 μM BAP or 0.1% DMSO prepared in DMEM supplemented with 5% FBS and 1% PS for 0.5, 1, 2, and 4 h.

Protein was collected after lysis with 1x cell lysis buffer (Cell Signaling Technology Inc (CST)) supplemented with 1% protease inhibitor cocktail (MilliporeSigma) and quantified with BCA protein assay kit (ThermoFisher Scientific). Total protein (25 μg) was separated on a 10% SDS-polyacrylamide gel and transferred to a PVDF membrane (Bio-Rad). The membranes were blocked in 5% milk dissolved in tris-buffered saline with tween-20 (TBS-T) for 1 h before overnight primary antibody incubation at 4 °C. Primary antibodies were diluted 1:1000 in 5% Bovine Serum Albumin (BSA) (Sigma–Aldrich) in TBS-T. Membranes were then washed and incubated for 1 h with secondary HRP-linked anti-rabbit antibody (CST, cat. # 7074) diluted 1:7500 in 5% milk in TBS-T imaged using the Signal Fire ECL Reagent (CST) on the iBright FL1500 (Thermofisher Scientific). The primary antibody was stripped with Restore PLUS western blot stripping buffer (Thermofisher Scientific) before probing for α-tubulin (CST, Cat. #2144) or β-actin (CST, cat. # 4970). Protein density was quantified using iBright Analysis Software.

Primary antibodies used in this experiment includes phospho-ERK1/2 (Thr202/Tyr204) (CST, cat. # 9101), ERK1/2 (CST, cat. # 4695), phospho-JNK (Thr183/Tyr185) (CST, cat. # 4668), JNK (CST, cat. # 9252), phospho-P38 (CST, cat. # 9211), P38 (CST, cat. # 9212), phospho-AKT (Ser473) (CST, cat. # 9271), AKT (CST, cat. # 9272), phospho-p70-S6K (CST, cat. # 9205), p70-S6K (CST, cat. # 9202), INSR (CST, cat. # 3025), and EGR1 (CST, cat. # 4154).

### 4.10 Immunocytochemistry (ICC)

mHypoE-46 cells were cultured in 8-well chamber slides in DMSM supplemented with 5% FBS and 1% P/S until 60% confluent. Cells were treated with 100 μM BAP or DMSO for 24 h, followed by ICC staining. The cells were washed with PBS and fixed with 4% paraformaldehyde (Electron Microscopy Sciences) in PBS for 10 min. Cells were then washed with PBS and permeabilized with 0.2% Triton X-100 in PBS (MilliporeSigma), followed by 2 h blocking with 5% BSA (MilliporeSigma) and 0.1% Triton X-100 in PBS and 2 h of primary antibody incubation at room temperature. Primary rabbit anti-mouse NPY antibody (Phoenix Pharmaceuticals Inc, H-049-03) was diluted 1:500 in antibody dilution buffer containing 1% BSA and 0.1% Triton X-100 in PBS. Control wells were incubated with antibody dilution buffer alone for 2 h. Cells were then washed with blocking buffer and incubated for 1 h with the Alexa Fluor 488 donkey anti-rabbit secondary antibody diluted 1:500 in antibody dilution buffer. After washing, the slides were mounted with the ProLong™ Gold Antifade Mountant with DAPI and sealed. Cells were visualized with a confocal laser-scanning microscope (Olympus LSM-GB200) at a 20× magnification. GFAP fluorescence was excited by the 488 nm argon laser line and DAPI was excited by the 408 nm laser line. Images were captured using the Zeiss Zen microscopy software.

### 4.11 Mouse phospho-RTK array

mHypoE046 cells were grown to 70–80% in DMEM supplemented with 5% FBS and 1% P/S. The cells were serum starved in plain 0% FBS DMEM for 1 h, followed by 5 min treatments with 100 μM BAP or 0.1% DMSO in DMEM supplemented with 0.1% FBS. Protein isolation and membrane incubation were performed as described in the manufacture protocol (R&D, #ARY014). The membranes were then imaged on the iBright FL1500 (Thermofisher Scientific) and protein density was quantified using iBright Analysis Software.

### 4.12 Chromatin immunoprecipitation (ChIP)

mHypoE-46 cells were grown to 70–80% and treated with 100 μM BAP or 0.1% DMSO for 0.5, 1, 2, and 4 h. An additional plate of cells was treated with DMSO (vehicle) per experimental replicate for immunoprecipication (IP) with the negative control (normal rabbit IgG) antibody. Cells were harvested with PBS containing 1x protease inhibitor cocktail. ChIP was performed using SimpleChIP Enzymatic Chromatin IP kit with magnetic beads (CST) according to the manufacturer’s instructions. IP was achieved using 10 μL of EGR1 antibody (CST, cat. # 4154) or 2 μL of normal rabbit IgG antibody (provided with the kit). DNA purification was conducted using PureLink PCR Purification Kit (Thermofisher Scientific). Binding of EGR1 to Npy 5′ upstream regulatory region was determined via RT-qPCR with cycling conditions as described above using primer specific for the Npy promotor (−1267 to −1165 relative to the transcriptional start site) designed based on previous EGR1 ChIP-seq analysis. Relative binding was calculated using the mean cycle of threshold (CT) of each IP sample and its respective 2% input sample with equation: % of input = 2% × 2(CT 2% input sample – CT IP sample).

### 4.13 Statistical analysis

Data was analyzed for statistical significance using GraphPad Prism (v.9.3.1) and R (v.4.3.0). All experiments represent biological replicates (n) of at least three independent experiments. ANOVA (three-way, two-way, and one-way) and multiple t-test were used when comparing the means of multiple groups with Bonferroni or respective post-hoc test as described in the figure legends. For indirect calorimetry studies, analysis of covariance (ANCOVA) was performed in R and CalR [128] for statistical analysis and then visualized in prism. Data are presented as means ± SEM with *P* values (for unpaired-t test) or adjusted *P* values (for all other tests) presented. *P*-values greater than 0.2 are presented as ns. On figures for daily measurement of body weight, food intake, and ipGTT, statistical significance was presented as ∗*P* < 0.05, ∗∗*P* < 0.01, ∗∗∗*P* < 0.001, ∗∗∗∗*P* < 0.0001. For all *in vivo* experiments, each replicate represents an individual mouse, except for food and water intake data for the male dose curve, male 2- and 4-week BAP, and female HFD/MFD cohorts, where each data point represents food/water intake per a cage of 2 mice. All replicates in *in vitro* experiments are individual biological replicates.

## CRediT authorship contribution statement

**Calvin V. Lieu:** Conceptualization, methodology, formal analysis, investigation, data curation, writing – original draft, visualization.

**Cindy X. Zhang:** Conceptualization, methodology, formal analysis, investigation, data curation, writing – original draft, visualization.

**Neruja Loganathan:** Writing – review & editing, methodology, investigation.

**Leon French:** Writing – review & editing, software, formal analysis.

**Andre Krunic:** Writing – review & editing, software.

**Sarah B. Cash:** Methodology, formal analysis, data curation.

**Jin Shi:** Methodology, formal analysis, data curation.

**Juliette Lee:** Writing – review & editing, methodology, investigation.

**Kacey J. Prentice:** Resources.

**Carolyn L. Cummins:** Writing – review & editing, supervision, resources, methodology, formal analysis, data curation.

**Jesse Gillis:** Writing – review & editing, resources, conceptualization.

**Denise D. Belsham:** Writing – review & editing, supervision, resources, visualization, project administration, funding acquisition, conceptualization.

## Disclosure statement

DDB has incorporated a company ZAPetite to house the BAP IP and conduct a phase 1 human trial. LF owns shares in Quince Therapeutics and has received consulting fees from PeopleBio Co., GC Therapeutics Inc., Cortexyme Inc., and Keystone Bio. All other authors have nothing to disclose.

## Funding

Canadian Institutes for Health Research (10.13039/501100000024CIHR).

Natural Sciences and Engineering Research Council (10.13039/501100000038NSERC).

Canada Foundation for Innovation, Connaught Innovation Fund at the 10.13039/501100003579University of Toronto, and 10.13039/501100001804Canada Research Chairs Program (DDB).

CVL was supported by the 10.13039/501100000064Banting and Best Diabetes Centre and NSERC CGS-M and CGS-D scholarships.

CXZ was supported by the Ontario Graduate Scholarship, 10.13039/501100000064Banting and Best Diabetes Centre and CIHR CGS-D scholarships.

## Declaration of competing interest

The authors declare the following financial interests/personal relationships which may be considered as potential competing interests: There is a patent pending for the compound named in this manuscript: PCT/CA2023/051000.

## Data Availability

Data will be made available on request.

## Abbreviations

AgRP: Agoutirelated peptide
ALPL: Alkaline phosphatase
AOC: Area of curve
Arc: Arcuate nucleus of the hypothalamus
AREG: Amphiregulin
ARE: Early growth response protein
BAP: 6benzylaminopurine
BRAF: VRaf Murine Sarcoma Viral Oncogene Homolog B
BTC: Betacellulin
CIDEA: Cell death inducing like effector a
ChIP: Chromatin immunoprecipitation
CMC: Carboxymethyl cellulose
COL1A1: Collagen alpha 1
DEG: Differentially expressed genes
DDIT4: DNA damage inducible transcript 4
DMSO: Dimethyl sulfoxide
EGFR: Epidermal growth factor receptor
EGR: Early growth response protein
EIF4EBP1: Eukaryotic translation inhibition factor 4Ebinding protein 1
EPA: United States Environmental Protection Agency
EREG: Epiregulin
ERK: Extracellular regulated kinase
GSEA: Gene set enrichment analysis
GO: Gene ontology
GOT: Glutamic oxaloacetic transaminase
GLP1R: Glucagon Like Peptide – 1 Receptor
HB-EGF: Heparin binding-EGF like growth factor
HFD: High fat diet
hiPSCHLN: Human induced pluripotent stem cell Hypothalamic like neurons
His3a: Histone 3a
iLINCS: Integrated Library of Integrated Networkbased Cellular Signatures
ipGTT: Intraperitoneal glucose tolerance test
JNK: cJun Nterminal kinases
LEP: Leptin
MAPK: Mitogen Activated Protein Kinase
MASLD: Metabolic dysfunctionassociated steatosis liver disease
MFD: 30% kilocalories from fat diet
mTORC1: Mammalian target of rapamycin complex 1
NPY: Neuropeptide Y
ORA: Overrepresentation analysis
P2R: P2 purinergic receptors
P38: P38 mitogen activated protein kinase
PGC1A: PPARG coactivator 1 alpha
PKA: Protein kinase A
POMC: Proopiomelanocortin
RNA-seq: RNA sequencing
Rpl7: 60s ribosomal protein L7
SESN2: Sestrin 2
TF: Transcription factor
TGFA: Transforming growth factor alpha
TSC2: Tuberous sclerosis complex 2
T2DM: Type 2 Diabetes Mellitus
UCP1: Uncoupling protein 1
ULK1: Unc 51 like kinase 1
WAT: White adipose tissue
