# Supplementary material for: The plant hormone, 6-benzylaminopurine, ameliorates obesity in male and female mice while on a high-fat diet
Source: Mol Metab. 2026 Apr 11;108:102366. doi: 10.1016/j.molmet.2026.102366 (PMC13133958; doi:10.1016/j.molmet.2026.102366)
Supplement: Multimedia component 1 [file mmc1.pdf]

Supplemental material for Lieu, Zhang et al.

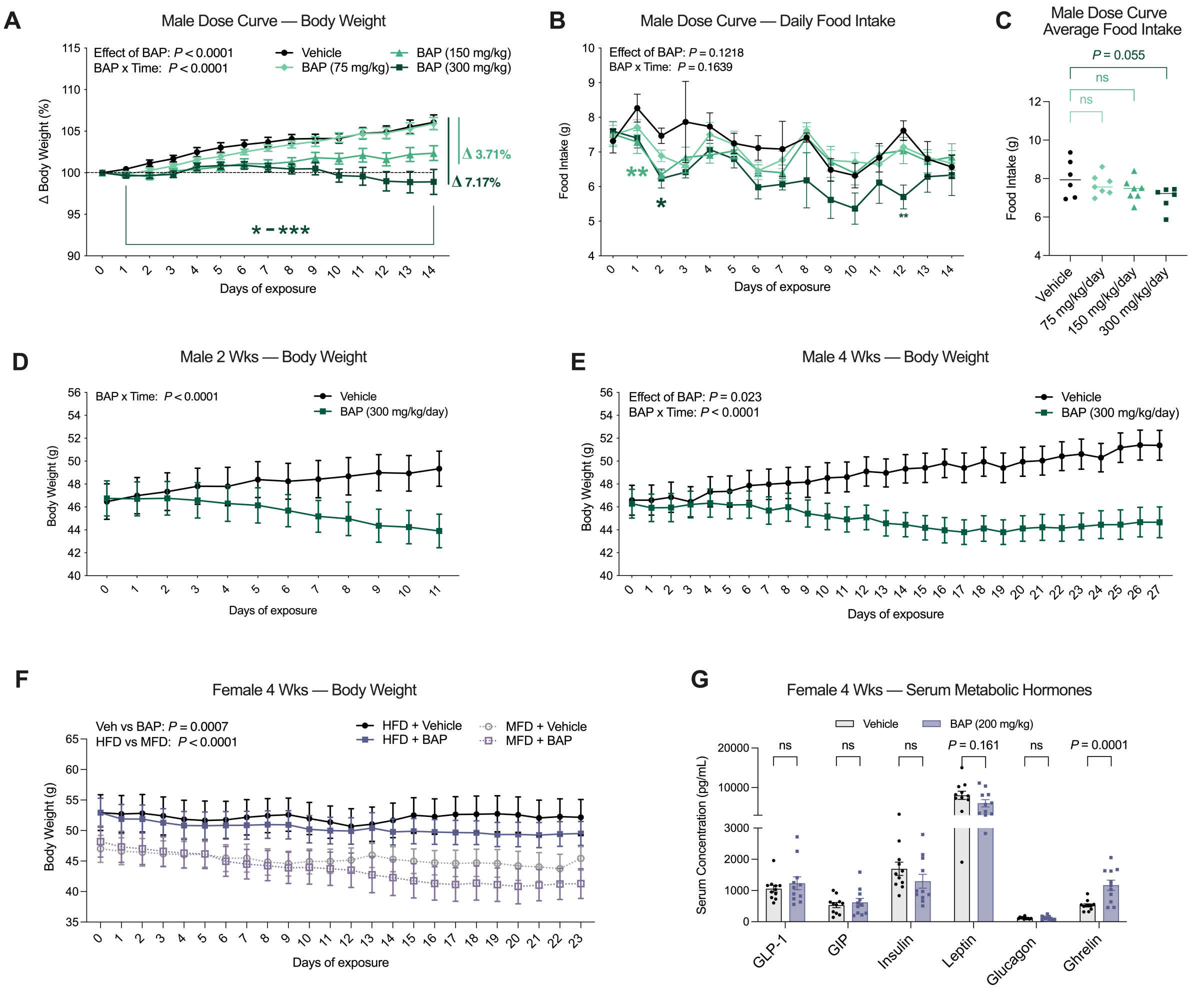

Fig. S1 BAP decreases body weight in male and female CD-1 mice on 60% HFD

A-C, Changes in **(A)** body weight as a percentage of starting weight, **(B)** daily food intake per cage, and **(C)** average daily food intake per cage of of male CD-1 mice fed with 75, 150, or 300 mg/kg/day BAP (n=14) or vehicle (n=13) emulsion for 2 weeks. **D, E,** Changes in body weight of male CD-1 mice subjected to **(D)** 2 weeks (n=10) and **(E)** 4 weeks (n=8) of daily 300 mg/kg BAP or vehicle emulsion feeding. **F,** Changes in body weight of female CD-1 mice fed with 4-week of 200 mg/kg/day BAP, with or without MFD intervention (n=12 for HFD groups, n=8-9 for MFD groups). **G,** Serum concentration of metabolic hormones of female CD-1 mice fed with BAP (200 mg/kg/day, 4 weeks) or vehicle emulsion without MFD intervention (n=11). Two-way ANOVA with Bonferroni post-hoc test**.** Values are presented as mean ± SEM with P values. P-values greater than 0.2 are presented as ns. Δ represent the net differences in body weight percentage between the BAP group and the vehicle control group. Data were analyzed using two-way ANOVA with Bonferroni post-hoc test for daily measurements of body weight **(A, B, D, E, F).** Unpaired two-tailed *t*-test was used for 2 group comparisons **(G).** Values are presented as mean ± SEM with P values. P-values greater than 0.2 are presented as ns.

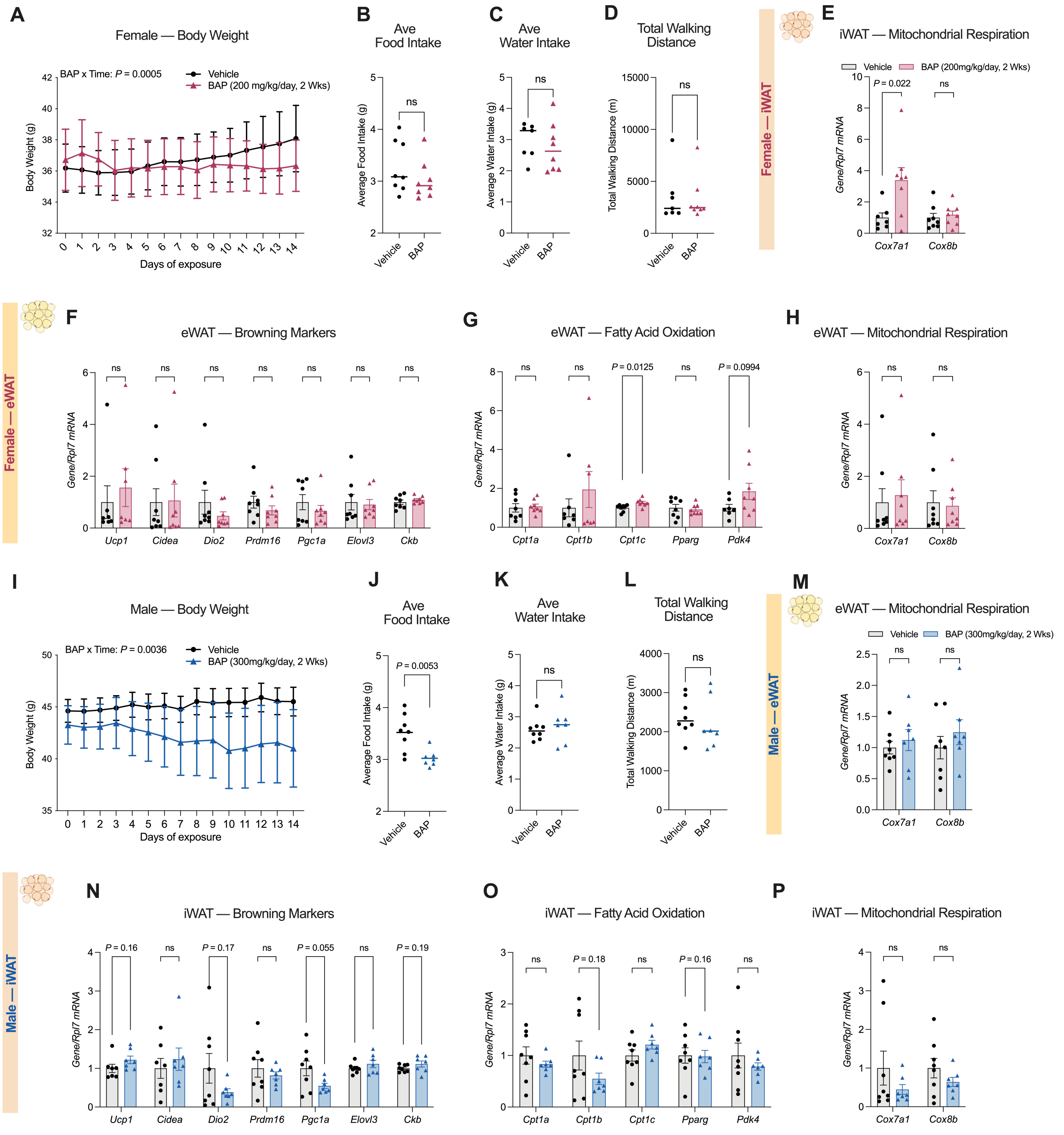

Q

	Sex	Dosage (mg/kg/day, Wks of exposure)	Diet while on BAP	Body Weight	Food Intake	Other Metabolic Changes
**Pilot Group**	Male	300, 2 Wks	60% HFD	↓	↓	Serum: ↓ Insulin ↓ Leptin ↑ GIP (*P* = 0.097) ↑ WAT browning ↓ MASLD markers
	Male	300, 4 Wks	60% HFD	↓	↓	Serum: ↑ GLP-1 (*P* = 0.12) ↑ GIP (*P* = 0.12) Improved GTT ↑ WAT browning ↓ MASLD markers
**Dose Curve Group**	Male	300, 2 Wks	60% HFD	↓	↓	NA
	Male	150, 2 Wks	60% HFD	NC	NC	NA
	Male	75, 2 Wks	60% HFD	NC	NC	NA
**Female HFD vs MFD**	Female	200, 4 Wks	60% HFD	↓	NC	Serum: ↑ Ghrelin
	Female	200, 4 Wks	30% MFD	↓	NC	NA
**Promethion Metabolic Cage Group**	Female	200, 2 Wks	60% HFD	↓	NC	↓ RER ↑ iWAT browning & FA oxidation markers ↑ Thermogenesis (*P* = 0.093)
	Male	300, 2 Wks	60% HFD	↓	↓	↓ RER (*P* = 0.064) ↑ eWAT *Ucp1*

Fig. S2 BAP differentially changes WAT function markers in female and male CD-1 mice

A-D, Changes in **(A)** body weight, **(B)** average daily food intake, **(C)** average daily water intake, and **(D)** total walking distance over the 2-week exposure period of female CD-1 mice fed 200 mg/kg/day BAP or vehicle emulsion for 2 weeks (n=8). **E,** Changes in the mRNA expression of mitochondrial respiration markers in iWAT of female mice fed BAP or vehicle emulsion for 2 weeks. **F-H,** Changes in the mRNA expression of markers for **(F)** browning, **(G)** fatty acid oxidation, and **(H)** mitochondrial respiration in eWAT of female mice fed BAP or vehicle emulsion for 2 weeks. **I-L,** Changes in **(I)** body weight, **(J)** average daily food intake, **(K)** average daily water intake, and **(L)** total walking distance over the 2-week exposure period of male CD-1 mice fed 300 mg/kg/day BAP or vehicle emulsion for 2 weeks (n=7-8). **M,** Changes in the mRNA expression of mitochondrial respiration markers in eWAT of male mice fed BAP or vehicle emulsion for 2 weeks. **N-P,** Changes in the mRNA expression of markers for **(N)** browning, **(O)** fatty acid oxidation, and **(P)** mitochondrial respiration in iWAT of female mice fed BAP or vehicle emulsion for 2 weeks. **Q,** A summary of the effects of BAP on body weight, food intake, circulating metabolic hormone, and other metabolic changes in male and female CD-1 mice. NC: No changes, NA: not tested.

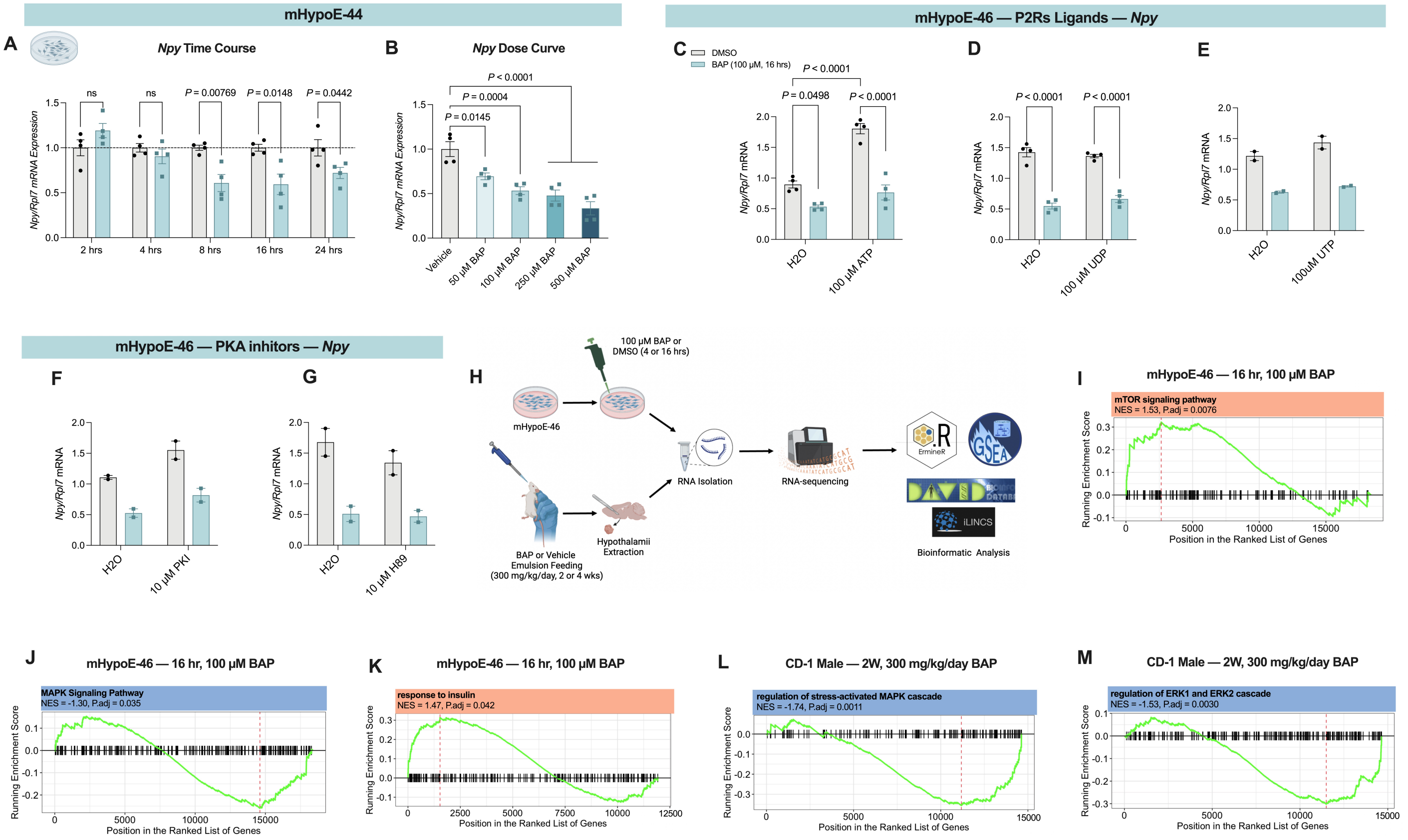

mHypoE-44
A
Npy Time Course
Npy/Rpl7 mRNA Expression
ns
ns
P = 0.00769
P = 0.0148
P = 0.0442
2 hrs
4 hrs
8 hrs
16 hrs
24 hrs
B
Npy Dose Curve
P < 0.0001
P = 0.0004
P = 0.0145
Vehicle
50 μM BAP
100 μM BAP
250 μM BAP
500 μM BAP
mHypoE-46 — P2Rs Ligands — Npy
C
DMSO
BAP (100 μM, 16 hrs)
P < 0.0001
P = 0.0498
P < 0.0001
Npy/Rpl7 mRNA
H2O
100 μM ATP
D
P < 0.0001
P < 0.0001
100 μM UDP
E
100uM UTP
mHypoE-46 — PKA inhitors — Npy
F
10 μM PKI
G
10 μM H89
H
100 μM BAP or DMSO (4 or 16 hrs)
mHypoE-46
RNA Isolation
RNA-sequencing
BAP or Vehicle Emulsion Feeding (300 mg/kg/day, 2 or 4 wks)
Hypothalamii Extraction
ErmineR
GSEA
iLINCS
Bioinformatic Analysis
I
mHypoE-46 — 16 hr, 100 μM BAP
mTOR signaling pathway
NES = 1.53, P.adj = 0.0076
Running Enrichment Score
Position in the Ranked List of Genes
J
mHypoE-46 — 16 hr, 100 μM BAP
MAPK Signaling Pathway
NES = -1.30, P.adj = 0.035
K
mHypoE-46 — 16 hr, 100 μM BAP
response to insulin
NES = 1.47, P.adj = 0.042
L
CD-1 Male — 2W, 300 mg/kg/day BAP
regulation of stress-activated MAPK cascade
NES = -1.74, P.adj = 0.0011
M
CD-1 Male — 2W, 300 mg/kg/day BAP
regulation of ERK1 and ERK2 cascade
NES = -1.53, P.adj = 0.0030

Fig. S3 Identifying the binding target of BAP receptor ligands and bioinformatic pathway analysis

A, Changes in *Npy* mRNA expression after 2 to 24 hour treatment with100 μM BAP in mHypoE-44 neuronal cell line (n=4). **B,** Changes in *Npy* mRNA expression after treatment of mHypoE-44 (n=4) cell line with increasing concentrations of BAP for 16 hours. **C-G,** Changes of *Npy* mRNA expression in mHypoE-46 neurons pre/cotreated with P2Rs ligands **(C)** 100 μM ATP, **(D)** 100 μM UDP, **(E)** 100 μM UTP, or PKA inhibitors, **(F)** 10 μM PKI, **(G)** 10 μM H89 or DMSO for 1 hours, followed by 16 hours with DMSO or 100 μM BAP (n=2-3). **H,** Experimental outline for RNA-seq samples preparations and bioinformatic analysis. **I-M,** Individual GSEA enrichment plots of **(I)** mTOR, **(J)** MAPK and **(K)** response to insulin pathways that are changed by BAP at 16 hours in mHypoE-46 neurons. **L, M,** Individual GSEA enrichment plots of **(L)** MAPK and **(M)** ERK1/2 cascades in the hypothalamii of male CD-1 mice subjected to 2 weeks of 300 mg/kg/day BAP. Data was analyzed using Two-way ANOVA with Bonferroni post-hoc test **(A, C, D).** One-way ANOVA with Bonferroni post-hoc test was used for the dose curve analysis **(B).** Values are presented as mean ± SEM with P values. P-values greater than 0.2 are presented as ns.

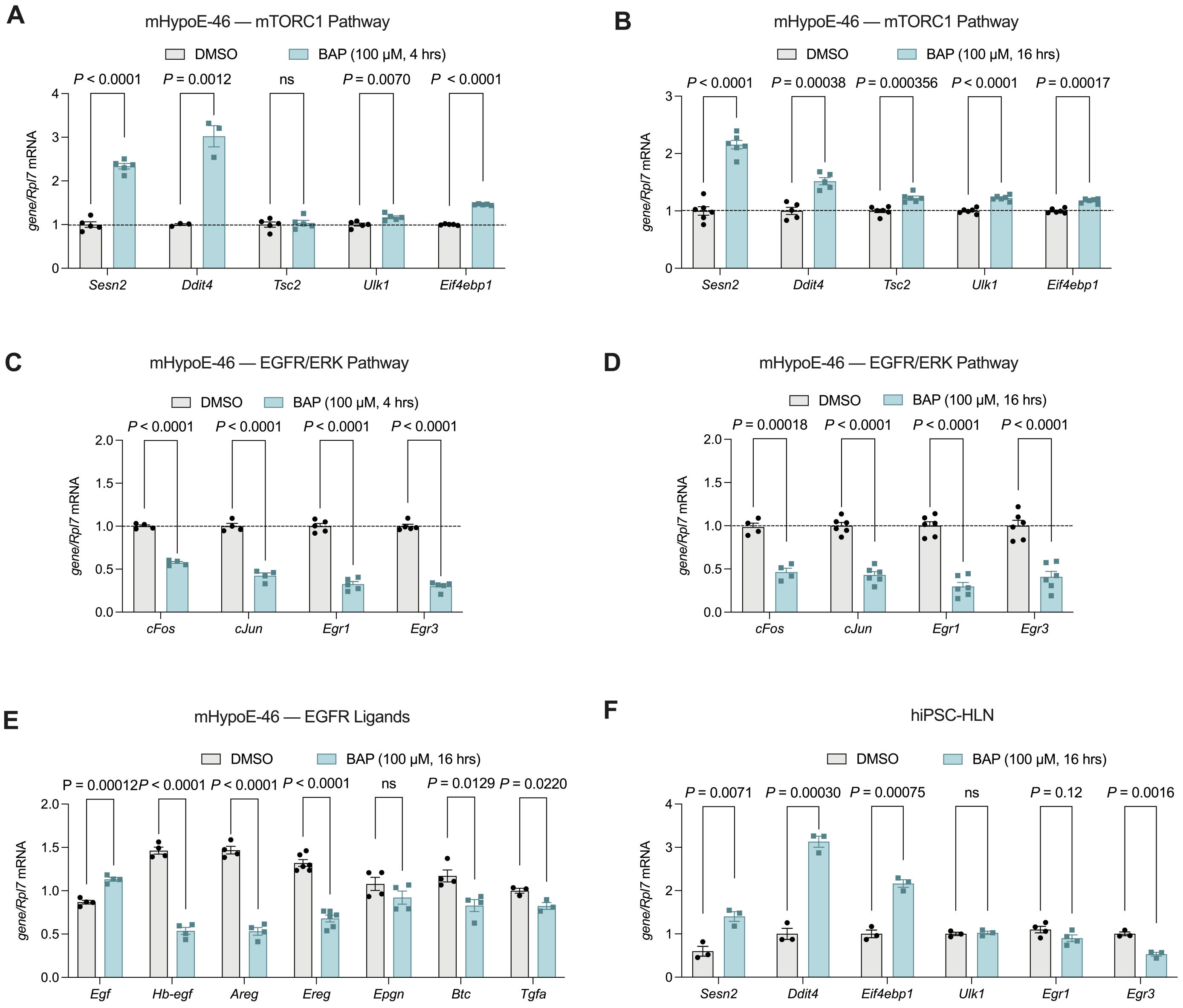
A
mHypoE-46 — mTORC1 Pathway
DMSO
BAP (100 μM, 4 hrs)
P < 0.0001
P = 0.0012
ns
P = 0.0070
P < 0.0001
gene/Rpl7 mRNA
Sesn2
Ddit4
Tsc2
Ulk1
Eif4ebp1
B
mHypoE-46 — mTORC1 Pathway
DMSO
BAP (100 μM, 16 hrs)
P < 0.0001
P = 0.00038
P = 0.000356
P < 0.0001
P = 0.00017
gene/Rpl7 mRNA
Sesn2
Ddit4
Tsc2
Ulk1
Eif4ebp1
C
mHypoE-46 — EGFR/ERK Pathway
DMSO
BAP (100 μM, 4 hrs)
P < 0.0001
P < 0.0001
P < 0.0001
P < 0.0001
gene/Rpl7 mRNA
cFos
cJun
Egr1
Egr3
D
mHypoE-46 — EGFR/ERK Pathway
DMSO
BAP (100 μM, 16 hrs)
P = 0.00018
P < 0.0001
P < 0.0001
P < 0.0001
gene/Rpl7 mRNA
cFos
cJun
Egr1
Egr3
E
mHypoE-46 — EGFR Ligands
DMSO
BAP (100 μM, 16 hrs)
P = 0.00012
P < 0.0001
P < 0.0001
P < 0.0001
ns
P = 0.0129
P = 0.0220
gene/Rpl7 mRNA
Egf
Hb-egf
Areg
Ereg
Epgn
Btc
Tgfa
F
hiPSC-HLN
DMSO
BAP (100 μM, 16 hrs)
P = 0.0071
P = 0.00030
P = 0.00075
ns
P = 0.12
P = 0.0016
gene/Rpl7 mRNA
Sesn2
Ddit4
Eif4ebp1
Ulk1
Egr1
Egr3

Fig. S4 BAP modulates mTROC1 and EGFR/ERK pathways in mouse and human hypothalamic neuronal models

A, B, The effect of **(A)** 4 hours and **(B)** 16 hours of 100 μM BAP treatment on the mRNA expression of genes in mTORC1 pathways (n=3-6). **C, D,** The effect of **(C)** 4 hours and **(D)** 16 hours of 100 μM BAP treatment on the mRNA expression of downstream effectors of EGFR/ERK pathway (n=4-6). **E,** The effect of 16 hours 100 μM BAP treatment on the mRNA expression of endogenous EGFR ligands (n=4). **F,** The effect of 16 hours 100 μM BAP treatment on genes in the mTORC1 pathway and EGFR/ERK cascade in hiPSC-HLN (n=3-4). Data was analyzed using unpaired two-tailed *t*-test. Values are presented as mean ± SEM with P values. P-values greater than 0.2 are presented as ns.

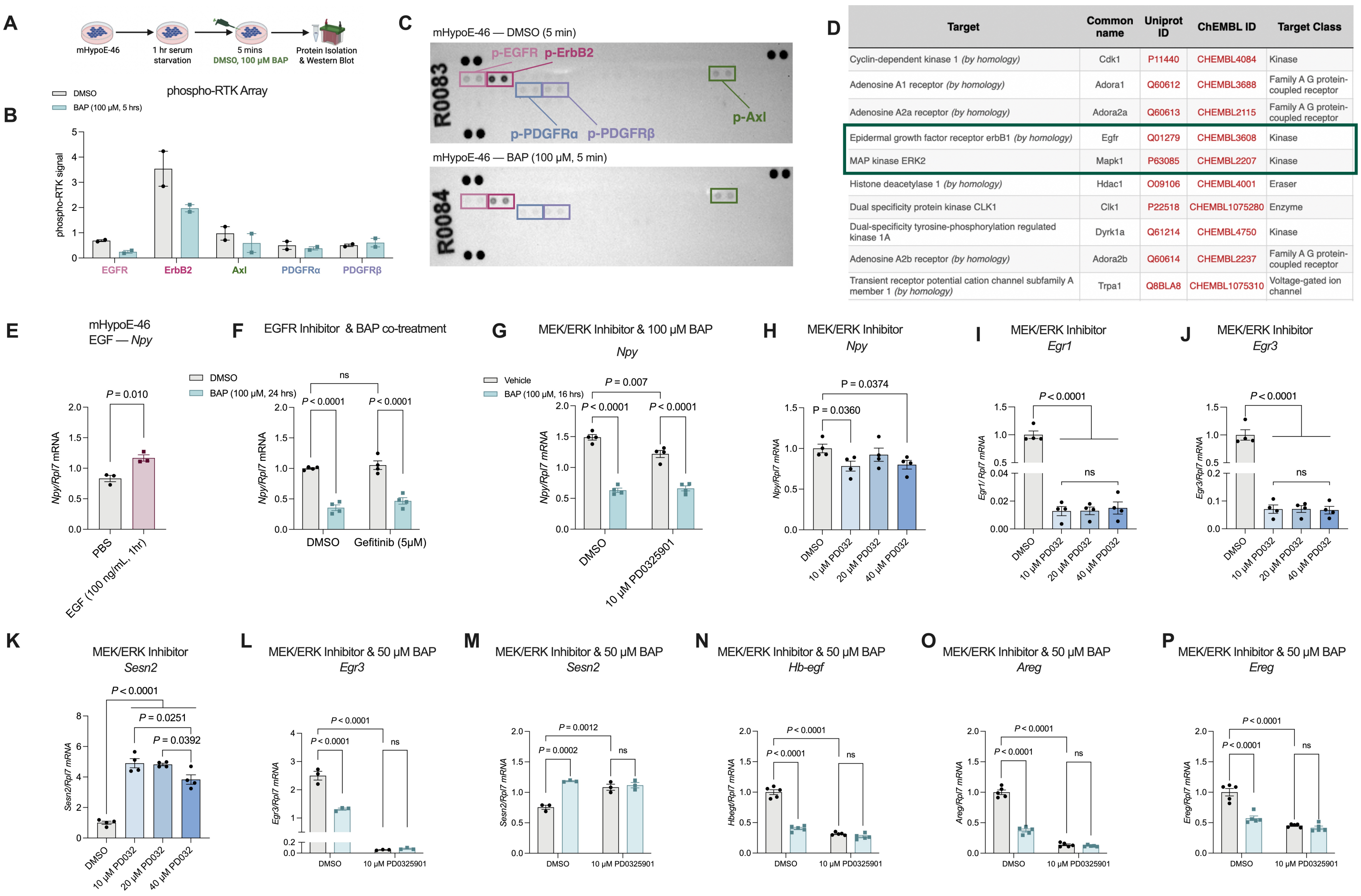

Target	Common name	Uniprot ID	ChEMBL ID	Target Class
Cyclin-dependent kinase 1 *(by homology)*	Cdk1	P11440	CHEMBL4084	Kinase
Adenosine A1 receptor *(by homology)*	Adora1	Q60612	CHEMBL3688	Family A G protein-coupled receptor
Adenosine A2a receptor *(by homology)*	Adora2a	Q60613	CHEMBL2115	Family A G protein-coupled receptor
Epidermal growth factor receptor erbB1 *(by homology)*	Egfr	Q01279	CHEMBL3608	Kinase
MAP kinase ERK2	Mapk1	P63085	CHEMBL2207	Kinase
Histone deacetylase 1 *(by homology)*	Hdac1	O09106	CHEMBL4001	Eraser
Dual specificity protein kinase CLK1	Clk1	P22518	CHEMBL1075280	Enzyme
Dual-specificity tyrosine-phosphorylation regulated kinase 1A	Dyrk1a	Q61214	CHEMBL4750	Kinase
Adenosine A2b receptor *(by homology)*	Adora2b	Q60614	CHEMBL2237	Family A G protein-coupled receptor
Transient receptor potential cation channel subfamily A member 1 *(by homology)*	Trpa1	Q8BLA8	CHEMBL1075310	Voltage-gated ion channel

Fig. S5 Identifying molecular mechanisms of BAP in hypothalamic neurons

A, Treatment outline for the mouse phosphor-RTK experiment. **B, C,** The effects of 5 minutes of 100 µM BAP or DMSO exposure on **(B)** RTK tyrosine phosphorylation levels with **(C)** representative membrane images (n=2). **D,** Top 10 putative binding targets of BAP predicted by SwissTargetPrediction in rodents. **E,** The effect of 1 hour 100 ng/mL EGF treatment on *Npy* expression in mHypoE-46 neurons (n=3). **F,** Changes of *Npy* mRNA expression in mHypoE-46 neurons pre/cotreated with EGFR inhibitor 5 µM gefitinib or DMSO for 1 hours, followed by 24 hours with DMSO or 100 µM BAP (n=4). **G,** Changes in *Npy* mRNA expression in mHypoE-46 neurons pre/cotreated with 10 µM MEK/ERK inhibitor PD0325901 or DMSO for 1 hours, followed by 16 hours with DMSO or 100 µM BAP (n=4). **H-K,** Changes in mRNA expression of **(H)** *Npy*, **(I)** *Egr1*, **(J)** *Egr3*, and **(K)** *Sesn2* in mHypoE-46 cells treated with a dose curve of PD0325901 or DMSO for 16 hours. **L-P,** Changes of **(L)** *Egr3*, **(M)** *Sesn2,* **(N)** *Hb-egf*, **(O)** *Areg*, and **(P)** *Ereg* mRNA expression in mHypoE-46 neurons pre/cotreated with 10 µM PD0325901 or DMSO for 1 hours, followed by 16 hours with DMSO or 50 µM BAP (n=3-5). Data was analyzed using Two-way ANOVA with Bonferroni post-hoc test **(E, G, L-P)**, One-way ANOVA with Bonferroni post-hoc test **(H-K)**, and unpaired two-tailed *t*-test **(E).** Values are presented as mean ± SEM with P values. P-values greater than 0.2 are presented as ns.

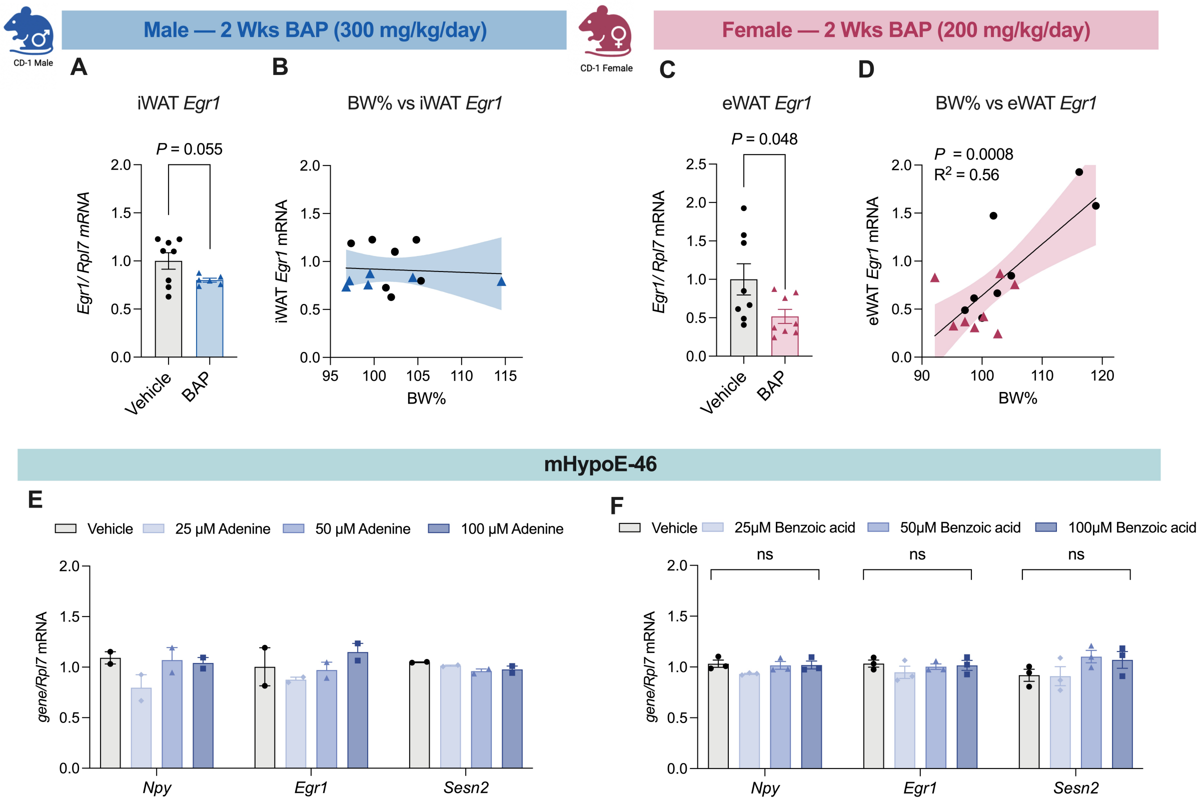
CD-1 Male
Male — 2 Wks BAP (300 mg/kg/day)
CD-1 Female
Female — 2 Wks BAP (200 mg/kg/day)
A
iWAT Egr1
P = 0.055
Egr1/ Rpl7 mRNA
Vehicle
BAP
B
BW% vs iWAT Egr1
iWAT Egr1 mRNA
BW%
C
eWAT Egr1
P = 0.048
Egr1/ Rpl7 mRNA
Vehicle
BAP
D
BW% vs eWAT Egr1
P = 0.0008
R2 = 0.56
eWAT Egr1 mRNA
BW%
mHypoE-46
E
Vehicle
25 μM Adenine
50 μM Adenine
100 μM Adenine
gene/Rpl7 mRNA
Npy
Egr1
Sesn2
F
Vehicle
25μM Benzoic acid
50μM Benzoic acid
100μM Benzoic acid
ns
gene/Rpl7 mRNA
Npy
Egr1
Sesn2

Fig. S6 BAP alters WAT *Egr1* expression in male and female CD-1 mice

A-D, Changes in WAT *Egr1* mRNA expression and its correlation with body weight change as a percentage of initial body weight in **(A, B)** male and **(C, D)** female CD-1 mice subjected to 2-week daily BAP feeding (male: 300 mg/kg/day, female: 200 mg/kg/day) (n=8). **E, F,** Changes of *Npy, Egr1,* and *Sesn2* mRNA expression in mHypoE-46 neurons treated with an increasing dose of **(E)** adenine and **(F)** benzoic acid. Data was analyzed using unpaired two-tailed *t*-test **(A, C)** and One-way ANOVA **(F)** with Bonferroni post-hoc test. Values are presented as mean ± SEM with P values. P-values greater than 0.2 are presented as ns.

Supplementary Table 1 – Primer sequences used in Lieu, Zhang et al.

Gene	Forward	Reverse	Product Length
Rpl7	TCG CAG AGT TGA AGG TGA AG	GCC TGT ACT CCT TGT GAT AGT G	114
Npy	CAG AAA ACG CCC CCA GAA	AAA AGT CGG GAG AAC AAG TTT CAT T	77
Agrp	CGG AGG TGC TAG ATC CAC AGA	AGG ACT CGT GCA GCC TTA CAC	69
Pomc	CCCGCCCAAGGACAAGCGTT	CTGGCCCTTCTTGTGCGCGT	112
Egr1	GCA CCT GAC CAC AGA GTC CTT	GCC ACA AAG TGT TGC CAC TG	158
Sesn2	GAACAACTCAGGGGGCTTTGA	TATCCAAATGCGGGGTCCTC	231
Ddit4	CTT CGT CCT CGT CTC GAA CT	CCA TCC AGG TAT GAG GAG TCT TCC	175
Tsc2	GAACTGAGTGGTGAATGCGG	CAAACGATCACCCTGTCCCT	210
Eif4ebp1	TCACTAGCCCTACCAGCGAT	ATTGTGACTCTTCACCGCCT	99
Ulk1	TCGACACCGCGAGAAGCA	AAGTCATACAGCGCCACGAT	144
Pik3r1	TATTGCGAGGGAAGCGAGAC	ACTTCGCCGTCTACCACTAC	91
Insr	TCC CAT CAA ATA TTG CCA AAA TT	CAG AAA TAG ATA AAT ACT TCC AAT CAC AAC AC	89
Areg	CGAAATGCCTTCTGGCAGTG	GGCTTAATCACCTGTTCAACTCTG	126
Btc	CAAACTCCCTCCTGCATCTGTG	GGTAAAACAGGTCCACTCGCT	70
Ereg	CGCTGCTTTGTCTAGGTTCCC	ACGGGGATCGTCTTCCATCT	125
Hb-egf	ACTGTCGTCCGTCTGTCTTC	GAAGTAGCCTCTGAAGGTTCCTAT	159
Egf	CTATCACTGCACATGCCCCA	GCGATGAACAACCAGTGCAA	194
Epgn	AACAACACCGAAGCTGACTAC	GCCAATCCCAATCGCAATGT	231
Egr3	TCA GAT GGC TAC AGA GAA TGT G	TTC CCA AGT AGG TCA CGG TC	122
cFos	CAACGAGCCCTCCTCCGACT	TGCCTTCTCTGACTGCTCACA	68
cJun	AATGGGCACATCACCACTAC	TGTTCTGGCTATGCAGTTCAG	124
Rpl7 (Human)	TGG CAA GAA AAG CTG GCA AC	ACC TTT CGA ACC TTT GGG CT	103
Npy (Human)	ATC AAC CTC ATC ACC AGG CAG	CAC CAC ATT GCA GGG TCT TC	128
His3a (Human)	GCAAGAGTGCGCCCTCTACTG	GGCCTCACTTGCCTCGTGCAA	218
Alpl	GCA CCT GCC TTA CCA ACT CT	GTG GAG ACG CCC ATA CCA TC	161

Supplementary Table 1 – Primer sequences used in Lieu, Zhang et al.

Ugt1a1	GCA GAG TGG TTT ATT CCC CCT AT	TTG ACA TAG GCT TCA AAT TCC TGG G	217
Got1	CAG GGA GAA TCG GGT TGG AG	CTC CGC ATC CCA GTA GCA AT	208
Got2	CCT AAA GCC AGA GCA GGT AGA G	CTG TTC CTT TGC ACC TGG GA	160
Col1a1	TGG TCC TGA TGG CAA AAC CG	CCT TTC CAG GTT CTC CAG CG	143
Ucp1*	TCTCAGCCGGCTTAATGACTG	GGCTTGCATTCTGACCTTCAC	80
Cidea	CAGAAATGGACACCGGGTAG	TGACATTGAGACAGCCGAGG	127
Dio2	TTCCTGGCGCTCTATGACTC	GTCAAGAAGGTGGCATTCGG	270
Pgc1a	ACTCTCAGTAAGGGGCTGGT	CCAGAGCAGCACACTCTATGT	157
Lep	GTTCCTGTGGCTTTGGTCCTA	GGATACCGACTGCGTGTGTG	133
Prdm16	GCCTTCACCGTTCTTCATGG	GACATCTGGGGGTGGAACAG	123
Elovl3	GCGTAAGCGTCCACTCATCT	CACCCGAAGGCACTTTGTTC	99
Ckb	AGCTGGCAGTAGAAGCTCTGTC	TCCGCCTCAGTCATGCTCTT	82
Adrb1*	CTCATCGTGGTGGGTAACGTG	ACACACAGCACATCTACCGAA	215
Adrb2*	ATGTCGGTTATCGTCCTGGC	GGTTTGTAGTCGCTCGAACTTG	81
Adrb3	CACCGCTCAACAGGTTTGAT	TTCTTGGGGCAACCAGTCAA	119
Th*	CCAAGGTTCATTGGACGGC	CTCTCCTCGAATACCACAGCC	137
Cox7a1	CAGGCTCTGGTCCGGTCTTT	AGGTCATTGTCGGCCTGGAAG	95
Cox8b	GTTCCCAAAGCCCATGTCTCT	CTGGAACCATGAAGCCAACGA	103
Cpt1a	TTGATCAAGAAGTGCCGGACGAGT	AAGCCTCATACGTGAGGCAGAACT	103
Cpt1b	TGGGCGACAGGCATTTTCTT	GGAGACGGACACAGATAGCC	127
Cpt1c	ACTTATGAGTCAGCCATGACCCGA	TGTCTCCTTGTTGTCCATGGCTCT	111
Pparg	GGTGACTTTATGGAGCCTAAG	CGGTCTCCACTGAGAATAATG	110
Pdk4	TGAACACTCCTTCGGTGCAG	GCCTTGAGCCATTGTAGGGA	173
Egr1 (ChIP)	GCCGCTTAGAATTGGGGTGT	TTTATGGAGCGCCCTTGTCG	161

*Primers sequences were from Tang Q, Liu Q, Li J, Yan J, Jing X, Zhang J, Xia Y, Xu Y, Li Y, He J. MANF in POMC Neurons Promotes Brown Adipose Tissue Thermogenesis and Protects Against Diet-Induced Obesity. Diabetes. 2022 Nov 1;71(11):2344-2359.
